# Electrochemical Reduction of CO_2_ to CO over Transition Metal/N‐Doped Carbon Catalysts: The Active Sites and Reaction Mechanism

**DOI:** 10.1002/advs.202102886

**Published:** 2021-10-31

**Authors:** Shuyu Liang, Liang Huang, Yanshan Gao, Qiang Wang, Bin Liu

**Affiliations:** ^1^ College of Environmental Science and Engineering Beijing Forestry University 35 Qinghua East Road, Haidian District Beijing 100083 P. R. China; ^2^ School of Chemical and Biomedical Engineering Nanyang Technological University 62 Nanyang Drive Singapore 637459 Singapore

**Keywords:** carbon‐based materials, carbon monoxide, CO_2_ reduction, single‐atom catalysts

## Abstract

Electrochemical CO_2_ reduction to value‐added chemicals/fuels provides a promising way to mitigate CO_2_ emission and alleviate energy shortage. CO_2_‐to‐CO conversion involves only two‐electron/proton transfer and thus is kinetically fast. Among the various developed CO_2_‐to‐CO reduction electrocatalysts, transition metal/N‐doped carbon (M‐N‐C) catalysts are attractive due to their low cost and high activity. In this work, recent progress on the development of M‐N‐C catalysts for electrochemical CO_2_‐to‐CO conversion is reviewed in detail. The regulation of the active sites in M‐N‐C catalysts and their related adjustable electrocatalytic CO_2_ reduction performance is discussed. A visual performance comparison of M‐N‐C catalysts for CO_2_ reduction reaction (CO_2_RR) reported over the recent years is given, which suggests that Ni and Fe‐N‐C catalysts are the most promising candidates for large‐scale reduction of CO_2_ to produce CO. Finally, outlooks and challenges are proposed for future research of CO_2_‐to‐CO conversion.

## Graphical abstract

The activity of M‐N‐C catalysts on CO_2_ reduction reaction (CO_2_RR) can be adjusted through regulation of active center and local atomic environments, including tuning the central metal atom and its neighbored coordinated atoms structure, or doping other heteroatoms and forming dual‐atoms sites. The role of nonmetal moieties and metal nanoparticles in M‐N‐C is also included.

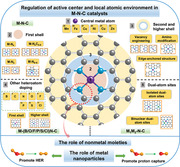

## 1 Introduction

Excessive utilization of fossil fuels has caused a severe energy crisis and a sharp rise in CO_2_ concentration in the atmosphere, which leads to global concern on the greenhouse effect.^[^ ^1^ ^]^ The Paris Agreement strives to achieve a “balance between anthropogenic emissions by sources and removals by sinks” in the second half of this century, which means that CO_2_ emitted to the atmosphere by human activity has to be eliminated either through nature‐based solutions (such as afforestation) or technological solutions that can store, capture and/or convert CO_2_. Especially, it is attractive to develop an effective approach to reduce CO_2_ into value‐added products, thereby this not only mitigates CO_2_ emission but also produces renewable chemicals/fuels to alleviate energy shortage.^[^ ^2^ ^]^ Over the past few decades, CO_2_ conversion technologies including thermocatalytic,^[^ ^3^ ^]^ photocatalytic,^[^ ^4^ ^]^ and electrocatalytic have been extensively studied. Electrocatalytic CO_2_ reduction to chemicals and fuels driven by renewable energy (e.g., wind, solar and geothermal energy) provides a promising approach for the carbon cycle.^[^ ^5^, ^6^ ^]^ It is also considered as an ideal strategy for storing renewable electricity in chemical bonds in addition to hydrogen production by water electrolysis. However, electrocatalytic reduction of CO_2_ still stays in its infancy as compared to water electrolysis technology.

Electrochemical CO_2_ reduction reaction (CO_2_RR) involves multiple electron/proton transfer processes, and CO_2_ can be reduced into various gaseous and liquid products including formic acid (HCOOH), carbon monoxide (CO), hydrocarbons (CH_4_ and C_2_H_4_), and alcohols (CH_3_OH and C_2_H_5_OH) through different pathways, which is dependent on the nature of electrocatalysts and the electrolytic conditions (e.g., applied potential, electrolyte, etc).^[^ ^7^ ^]^ The first step for CO_2_ activation to generate the CO_2_•^‐^ radical intermediate is difficult without a catalyst.^[^ ^8^ ^]^ But with the help of an electrocatalyst, the CO_2_•^‐^ radical can be stabilized through a chemical bond generated between CO_2_ and the electrocatalyst, resulting in a less negative redox potential. Generally, selective production of desired products with high efficiency as well as high selectivity is desirable. Among the various CO_2_RR products, only CO and HCOOH production have achieved remarkable selectivity close to 100%, showing the great potential for industrial application.^[^ ^9^, ^10^, ^11^, ^12^ ^]^ Furthermore, as the main component of syngas, CO is a crucial raw material along with H_2_ for the Fischer‐Tropsch synthesis.^[^ ^13^ ^]^ Notably, despite the equilibrium potential for CO_2_ reduction to CO is low (‐0.11 V vs RHE at pH = 7), a higher overpotential is typically required to overcome the sluggish reaction kinetics.^[^ ^14^ ^]^ The high overpotential would also cause an enhanced hydrogen evolution reaction (HER), competing with the CO_2_RR, which lowers the CO selectivity.^[^ ^15^ ^]^ Therefore, the development of effective electrocatalysts is highly critical to reduce the overpotential and suppress HER to achieve high selectivity and activity in CO_2_RR.

Recently, several types of electrocatalysts for efficient CO_2_‐to‐CO conversion have been reported, including noble metals (e.g., Au, Ag, Pd), Zn as well as carbon‐based materials,^[^ ^14^, ^16^, ^17^, ^18^ ^]^ among which, single‐atom catalysts (SACs) have attracted great attention.^[^ ^19^ ^]^ Different from conventional nanocatalysts, SACs downsize to atomic level that can boost the exposed active sites and achieve a maximum atom utilization.^[^ ^20^, ^21^ ^]^ In SACs, the supporting substrates, e.g., carbon‐based materials, are not only used to anchor single metal atoms but also used to tune the charge distribution and electronic structure of the metal atoms. Besides, for the carbon‐rich supports, doping N into the carbon matrix to introduce additional defects offers an efficient way to anchor single metal sites and tune the electronic structure of the carbon surface. Some reports used M‐N‐C to represent the carbon‐based catalysts containing atomically dispersed M sites anchored on N‐doped carbon support,^[^ ^19^ ^]^ which show great potential for electrochemical CO_2_‐to‐CO reduction. M‐N‐C catalysts for CO_2_ electroreduction is becoming highly attractive in recent years because of their high CO selectivity, good stability, and low cost. Recently, some reviews on the use of SACs for CO_2_ electroreduction have been summarized from the aspects of catalyst preparation, characterization, and performance,^[^ ^22^, ^23^, ^24^, ^25^ ^]^ while some other reviews focus on reviewing different types of catalysts including metal‐based and carbon‐based electrocatalysts for CO_2_ electroreduction to CO.^[^ ^14^, ^16^ ^]^ However, so far there is no comprehensive review of different coordination structures of transition metal/N‐doped carbon (M‐N‐C) catalysts that have been reported for CO_2_RR. Herein, experimental and theoretical investigations of M‐N‐C catalysts are summarized here to understand the effect of the active center and the local atomic environments on activity and selectivity, as well as the role of nonmetal moieties and metal nanoparticles in M‐N‐C in electrochemical CO_2_RR. A visual performance comparison of M‐N‐C catalysts with different central metal atoms for CO_2_ reduction reaction (CO_2_RR) reported over the recent years is also given.

## 2 Reaction Mechanism of Electrochemical CO_2_ Reduction to CO

**Figure 1:**
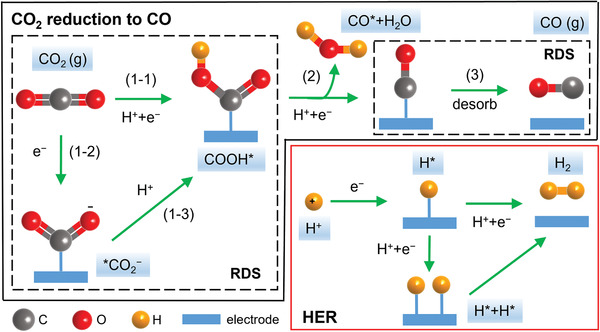
Proposed reaction pathway for CO_2_ reduction to CO and HER (C atoms: gray; O atoms: red; H atoms: orange; and electrode: blue).

**Figure** **1** displays the possible reaction pathway for CO_2_ electroreduction to CO and hydrogen evolution reaction (HER).^[^ ^26^ ^]^ The concerted proton‐electron transfer (CPET) reaction (Equation 1‐1) is usually considered as the first step toward CO production. Besides, a proton decoupled electron transfer process is proposed, where *COOH is formed through two steps (Equations 1–2 and 1–3). Previous studies suggested that the rate‐determining step (RDS) could be the reaction (1‐1), (1‐2), or (3), depending on the binding energy of CO_2_^‐^* and COOH* as well as the desorption energy of CO*. Therefore, it is critical to tune the adsorption strength of the key intermediates to develop ideal electrocatalysts for CO_2_ reduction to CO, which should strongly bind *COOH but weakly bind *CO so that it could promote both the *COOH formation and the *CO desorption steps. However, the adsorption of COOH* and CO* are usually linearly related, especially on transition metals.^[^ ^27^ ^]^ Therefore, the binding energy of COOH* (or CO*) was used as a descriptor for CO_2_RR to CO, and some studies have shown a volcano relationship between adsorption energy of COOH* (or CO*) and catalytic activity.^[^ ^26^, ^28^ ^]^ In addition to CO_2_RR intermediates, hydrogen adsorption on the catalyst's surface also needs to be considered since HER is the major competing reaction to CO_2_RR. Strong binding of *H is desired as it can lead to a large overpotential in HER and facilitate CO_2_ electroreduction.

(1‐1) $$CO_{2}\left(g\right)+*+H^{+}\left(\mathrm{aq}\right)+e^{-}\to \mathrm{COOH}*$$

(1‐2) $$CO_{2}\left(g\right)+*+e^{-}\to C{O_{2}}^{-}*$$

(1‐3) $$C{O_{2}}^{-}*+H^{+}\to \mathrm{COOH}*$$

(2) $$\mathrm{COOH}*+H^{+}\left(\mathrm{aq}\right)+e^{-}\to \mathrm{CO}*+H_{2}O$$

(3) $$\mathrm{CO}*\to \mathrm{CO}\left(g\right)+*$$

Over the past decades, many novel electrocatalysts have been reported for selective CO_2_‐to‐CO conversion. Noble metal‐based electrocatalysts such as Au,^[^ ^29^ ^]^ Ag,^[^ ^30^ ^]^ and Pd^[^ ^31^ ^]^ were regarded as one of the major classes for efficient CO_2_RR to CO with high selectivity (>80%) and activity because they could stabilize *COOH intermediate. However, for further industrial applications, low‐cost and earth‐abundant electrocatalysts with excellent performance need to be exploited to replace the precious metals. Some non‐noble metal‐based catalysts like Zn,^[^ ^32^ ^]^ bimetallic Cu–Sn^[^ ^33^ ^]^ and Cu–In^[^ ^34^ ^]^ were found capable of selectively reducing CO_2_ to CO. Recently, a new class of heteroatom‐doped carbon‐based materials,^[^ ^35^, ^36^, ^37^ ^]^ have been shown very active in CO_2_RR to CO in aqueous solution. For the metal‐free N‐doped carbon‐based catalysts, the pyridinic‐N and/or graphitic‐N defects were identified as the active sites for CO_2_ reduction.^[^ ^38^, ^39^, ^40^, ^41^ ^]^ Notably, the binding energies of *COOH and *CO intermediates on the N‐doped sites no longer follow the linear scaling relationship that generally appears in pure transition metals, which is possibly due to the heterogeneity caused by the N dopants into the carbon matrix.^[^ ^38^ ^]^ Furthermore, inspired by heterogeneous organometallic complexes with high activity toward CO_2_ electroreduction,^[^ ^42^, ^43^, ^44^ ^]^ introducing single transition metal sites into N‐doped carbon matrix to obtain heterogeneous M‐N‐C electrocatalysts, was observed capable of significantly improving the current density and CO selectivity.^[^ ^45^, ^46^, ^47^ ^]^ For example, Strasser et al.^[^ ^48^ ^]^ reported that introducing metal centers (Fe or Mn) into N‐doped carbon matrix could significantly enhance the CO_2_RR activity in contrast with metal‐free N‐doped carbon catalysts and the single M‐N*_x_* sites were regarded as the dominant active centers responsible for high CO_2_‐to‐CO activity.

## 3 Regulation of Active Center and Local Atomic Environment in M‐N‐C Catalysts

**Figure 2:**
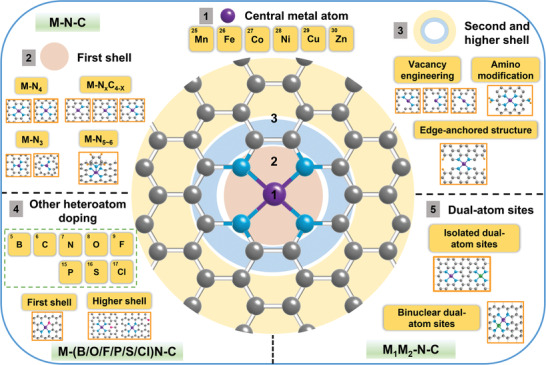
Schematic illustration showing regulation of central metal atom and local atomic environment in M‐N‐C catalysts (C atoms: gray; N atoms: blue; and metal atoms: purple).

M‐N‐C catalysts are typically synthesized through high‐temperature pyrolysis (usually above 700 °C), during which precursor containing a transition metal, C (and N) was decomposed under inert gas (i.e., N_2_, Ar) or NH_3_ gas atmosphere. During the high‐temperature treatment, the metal center was coordinated with N atoms to form active M‐N*_x_* sites, whereas the structures were usually uncontrolled. Heterogeneous molecular M‐N‐C catalysts with well‐defined M−N_4_ moieties could be obtained via attaching metallomacrocycles onto carbon supports.^[^ ^49^, ^50^, ^51^ ^]^ The central metal atom and the coordination environment have a significant effect on the intrinsic electrocatalytic activity of M‐N‐C catalysts in CO_2_RR (**Figure** **2**). The coordination environment includes the first shell, second shell, and even higher shell of the metal center.

Here we divided the regulation strategies into five classes, including regulation of central metal atom, engineering the first shell of the metal coordination environment, engineering the second and higher shell of the metal coordination environment, other heteroatom doping, and establishing dual‐atom site catalysts. The regulation of the central metal atom is easy to be operated by changing metal salt precursor, which is closely related to the main CO_2_RR products. In contrast to the central atoms, the surrounding coordination atoms own a broader regulation range. Currently, most studies focused on engineering the first shell of the metal coordination environment, and the approach of accurately synthesizing atomically dispersed M‐N_4_ sites has been developed. However, the accurate synthesis of a more compact coordination structure is still an obstacle due to the inhomogeneity caused by the high temperature used in the preparation of M‐N‐C catalysts. In addition to C or N coordinated atoms in M‐N‐C, other heteroatom doping provides more opportunities to adjust the electronic structure of the central atoms. Establishing dual‐atom sites offers another way to reduce the barriers for the key reaction intermediates formation in CO_2_RR and may be promising for syngas or C_2+_ products production.

### 3.1 Regulation of Central Metal Atom

#### 3.1.1 Activity Comparison of Different Metal Centers

**Figure 3:**
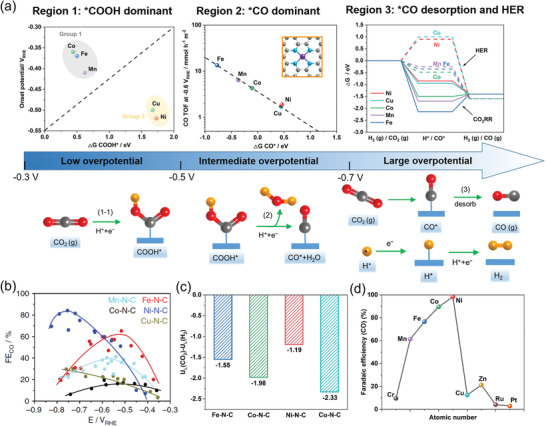
M‐N‐C catalysts with different central metal atoms for CO_2_‐to‐CO conversion. a) Three potential regions with distinctly different rate‐determining mechanistic features for M‐N‐C (M = Mn, Fe, Co, Ni, Cu) catalysts. b) CO FE versus applied potentials for M‐N‐C catalysts (M = Mn, Fe, Co, Ni, Cu). c) The values of U_L_(CO_2_)‐U_L_(H_2_) for all M‐N‐C catalysts. d) CO FE for M‐N‐C at ‐1.2 V versus RHE. a,b) Reproduced with permission.^[^ ^47^ ^]^ Copyright 2017, Springer Nature. c) Reproduced with permission.^[^ ^55^ ^]^ Copyright 2020, WILEY‐VCH. d) Reproduced with permission.^[^ ^56^ ^]^ Copyright 2019, Springer Nature.

The CO_2_RR selectivity largely relies on the nature of the metal center in M‐N‐C electrocatalysts. The central metal atoms in M‐N‐C materials reported for CO production include Mn, Fe, Co, Ni, Cu, and Zn. Strasser et al.^[^ ^47^ ^]^ developed a variety of M‐N‐C eletrocatalysts (M = Mn, Fe, Co, Ni, Cu) for CO_2_ electroreduction to explore the influence of the central metal atom. DFT calculations (**Figure** **3a**) employing typical M‐N_4_ sites (Figure 3b) indicated that the M‐N‐C electrocatalysts could be classified into two groups: Fe, Mn, and Co‐N‐C catalysts with strong binding to *COOH requiring low overpotentials for CO_2_‐to‐CO conversion, and Cu and Ni‐N‐C catalysts with weak binding to *COOH requiring considerably higher overpotentials. Moreover, the Ni‐N‐C with weak binding to H* inhibited HER, while the Fe, Mn, Co‐N‐C with stronger binding of H* displayed a higher HER activity in the large potential range, leading to a lower CO selectivity. Notably, Cu‐N‐C is an exception because of the thermodynamic instability of Cu‐N*_x_* sites at large overpotentials. Consequently, the CO selectivity in the whole potential range follows: Ni‐N‐C > Fe‐N‐C > Mn‐N‐C > Cu‐N‐C ≈ Co‐N‐C (Figure 3b). Considering the trade‐off between energy input and product yield, the Ni‐N‐C catalyst achieving the highest CO selectivity at high overpotentials and the Fe‐N‐C catalyst achieving relatively high CO selectivity at low overpotentials are the most promising candidates. Other researchers obtained similar results.^[^ ^52^, ^53^, ^54^ ^]^ It is noted that most of the M‐N‐C electrocatalysts are prepared by a high‐temperature pyrolysis approach, it is hard to rule out the effect of the surrounding coordinating atoms when comparing different metal active centers. Jiang et al.^[^ ^55^ ^]^ synthesized a series of M‐N‐C (M = Ni, Fe, Co, Cu) catalysts from multivariate metal‐organic frameworks (MTV‐MOFs) with similar metal coordination environments (pyridinic‐type M‐N_4_), which provided an ideal model for the investigation of the intrinsic activities of various single metal atoms for CO_2_RR. Among the synthesized catalysts, Ni‐N‐C achieved a maximum CO selectivity of 96.8%, followed by Fe‐N‐C (86.5%), Cu‐N‐C (14.0%), and Co‐N‐C (17.8%). DFT calculations were performed and the limiting potential difference between CO_2_RR and HER (U_L_(CO_2_)‐U_L_(H_2_)) was calculated (Figure 3c), used as a descriptor for product selectivity, which was well consistent with the experimental results. Moreover, a universal ligand‐mediated approach for transition metal (including Cr, Mn, Fe, Co, Ni, Cu, Zn, Ru, Pt) single‐atom catalysts synthesis containing MN_4_ sites over carbon support was developed.^[^ ^56^ ^]^ Interestingly, a volcano relation between CO FE and metal atomic number was observed (Figure 3d), which might be related to the d‐band center of metal in the M‐N‐C catalysts.

**Figure 4:**
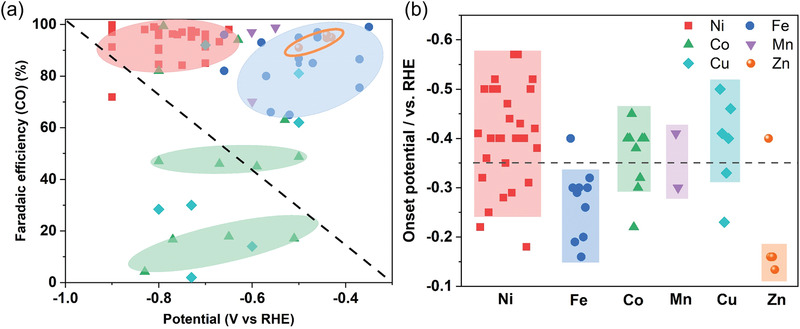
a) Reported maximum CO FEs and b) onset potentials for various M‐N‐C catalysts (M = Mn, Fe, Co, Ni, Cu, Zn). The point at the upper right of the dotted line in Figure 4a indicates that high CO selectivity can be achieved at a low applied potential. Data displayed in this figure are obtained from Tables S1–S6 (Supporting Information).

The CO_2_RR performance of the recently reported M‐N‐C catalysts with different central metal atoms is summarized in Tables S1–S6 (Supporting Information) and **Figure** **4**. As displayed in Figure 4, the CO selectivity and onset potential of different M‐N‐C catalysts are compared. Ni‐N‐C, Fe‐N‐C and Zn‐N‐C catalysts are the most promising candidates for large‐scale CO_2_ conversion to produce CO. The Ni‐N‐C catalysts achieved maximum CO FEs over 90% in the potential range from ‐0.7 to ‐0.9 V versus RHE, while Fe‐N‐C reached an average CO FE of ≈80% under lower overpotentials. Notably, the Zn‐N‐C catalysts, which are rarely reported in contrast with Ni‐N‐C and Fe‐N‐C, display the lowest onset potential and tend to reach high CO selectivity at low overpotentials. For instance, a Zn‐N‐G catalyst, in which the single Zn atomic site was coordinated with four N atoms on graphene, exhibited a maximum CO selectivity up to 91% at ‐0.5 V.^[^ ^57^ ^]^ DFT calculation suggested that the Zn‐N*_x_* sites facilitated COOH* intermediate formation and CO* desorption, leading to enhanced activity for CO_2_ electroreduction. As atomically dispersed Zn sites showed excellent activity for CO_2_‐to‐CO conversion, it is crucial to exclude the existence of atomically dispersed Zn sites when Zn‐based MOF materials were used as precursors to prepare M‐N‐C catalysts for CO_2_RR. Other M‐N‐C catalysts (M = Co, Cu, Mn) displayed quite different CO FEs for distinct samples, some of which showed comparable selectivity to Ni and Fe‐N‐C, while others exhibited lower values. This distinction may be attributed to the different coordination environments of central atoms. Despite Cu‐N‐C catalysts showed low selectivity for CO_2_ to CO production, they deserve more research attention as they have the potential to reduce CO_2_ to multi‐carbon products.^[^ ^58^, ^59^, ^60^ ^]^

#### 3.1.2 Activity Comparison of Ni SACs

**Figure 5:**
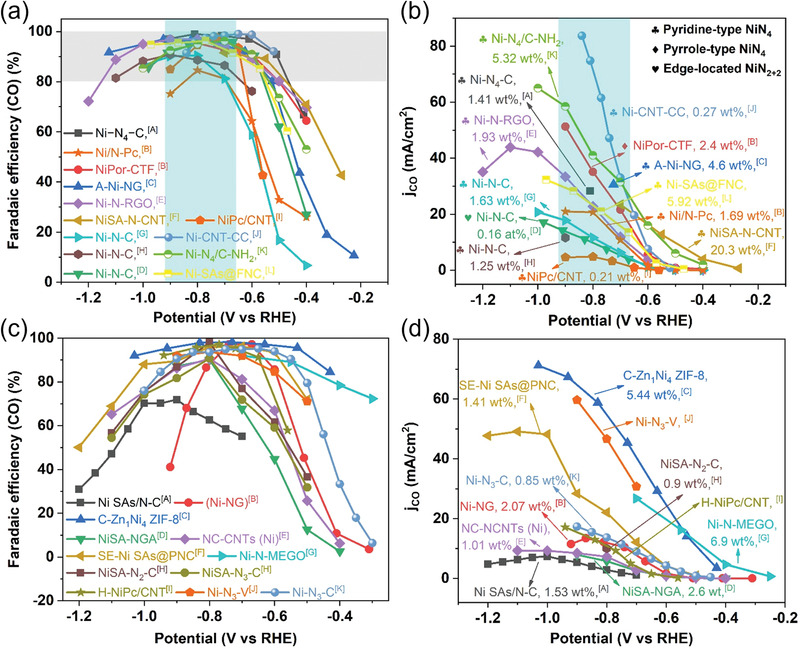
Catalytic activity comparison for various previously reported Ni‐N‐C catalysts with NiN_4_ moieties and NiN*_x_* (*x* < 4) moieties. a) CO FE and b) *j*_CO_ versus applied potential for Ni‐N‐C catalysts with NiN_4_ moieties. The data were obtained from the following studies: [A] Li et al.;^[^ ^10^ ^]^ [B] Lu et al.;^[^ ^61^ ^]^ [C] Yang et al.;^[^ ^62^ ^]^ [D] Pan et al.;^[^ ^63^ ^]^ [E] Jeong et al.;^[^ ^64^ ^]^ [F] Zhao et al.;^[^ ^65^ ^]^ [G] Zheng et al.;^[^ ^66^ ^]^ [H] Yuan et al.;^[^ ^67^ ^]^ [I] Sa et al.;^[^ ^68^ ^]^ [J] Liu et al.;^[^ ^50^ ^]^ [K] Chen et al.;^[^ ^69^ ^]^ [L] Han et al..^[^ ^70^ ^]^ c) CO FE and b) *j*_CO_ versus applied potential for Ni‐N‐C catalysts with NiN*_x_* (*x* < 4) moieties. The data were obtained from the following studies: [A] Zhang et al.;^[^ ^71^ ^]^ [B] Jiang et al.;^[^ ^72^ ^]^ [C] Yan et al.;^[^ ^73^ ^]^ [D] Mou et al.;^[^ ^74^ ^]^ [E] Fan et al.;^[^ ^75^ ^]^ [F] Yang et al.;^[^ ^76^ ^]^ [G] Cheng et al.;^[^ ^77^ ^]^ [H] Gong et al.;^[^ ^78^ ^]^ [I] Sa et al.;^[^ ^68^ ^]^ [J] Rong et al.;^[^ ^79^ ^]^ [K] Zhang et al.^[^ ^80^ ^]^

Among various M‐N‐C electrocatalysts, Ni‐N‐C catalysts have shown the best performance and drawn great attention for electrochemical CO_2_ reduction to CO. Therefore, we further compared the CO_2_RR performance of some recently reported Ni‐N‐C catalysts with different Ni‐N*_x_* sites (**Figure** **5**). It can be observed that in all cases CO FE follows a similar trend (Figure 5a), achieving a maximum value higher than 80% in the potential of ‐0.7–‐0.9 V for Ni‐N‐C catalysts with NiN_4_ moieties. Figure 5c compares the CO_2_RR activity of the Ni SACs with Ni coordination number to N lower than 4. Most of these catalysts exhibit high CO FEs over 90%, similar to those Ni‐N‐C catalysts with dominant NiN_4_ moieties. But they seem to reach the maximum CO selectivity under different potential ranges, which may be due to the different coordination environments of the central metal atom. For the purpose of practical application, CO partial current density (*j*_CO_) should be the main consideration as it represents the product yield. In addition to improving the intrinsic reactivity of active centers, increasing the density of active sites in the Ni‐N‐C catalysts can also effectively enhance the electrocatalytic efficiency. Nevertheless, it is notable that the Ni content is not always proportional to the *j*_CO_ in different Ni‐N‐C electrocatalysts, which may be due to the fact that some single Ni atoms are encapsulated within the carbon matrix that are inaccessible to CO_2_ molecules. The future direction towards improving the *j*_CO_ should maximize exposure of active single metal sites at the catalyst's surface.

### 3.2 Engineering the First Shell of the Metal Coordination Environment

The first shell represents the surrounding atoms that directly bond to the central metal atom, which can be adjusted by tuning the coordination number or changing the chemical identity of neighboring atoms. In this section, we mainly introduce M‐N‐C catalysts with typical M‐N_4_ structure, M‐N_3_ structure, M‐N_5–6_ structure and M‐N*_x_* C_4‐_ *_x_* (*x* = 1–3) structures for electrochemical CO_2_ reduction.

#### 3.2.1 M‐N_4_ Moieties

**Figure 6:**
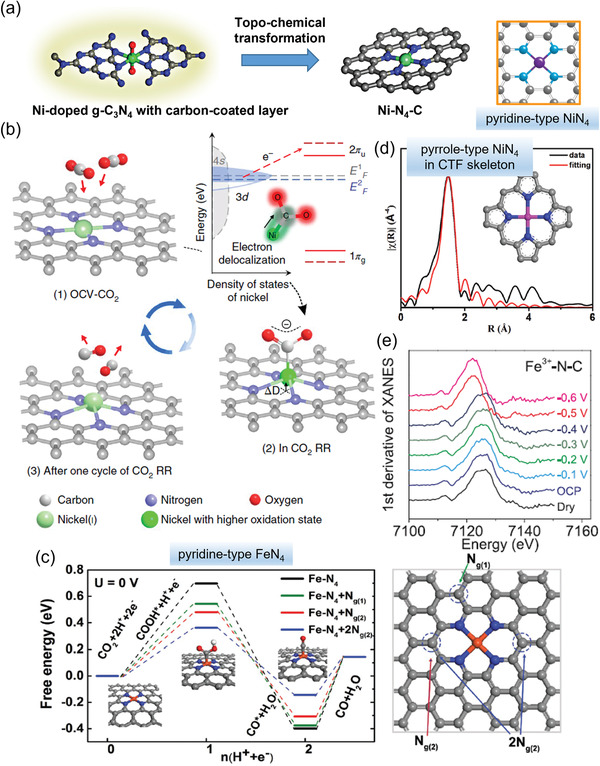
M‐N‐C with different MN_4_ sites for CO_2_‐to‐CO conversion. a) Schematic illustration showing the topo‐chemical transformation strategy for Ni‐N_4_‐C synthesis (Ni atoms, green; N atoms, blue; C atoms, gray; O atoms, red). b) Structural evolution of the Ni(I) active site in electrochemical CO_2_ reduction. c) Calculated Gibbs free energy diagrams for CO_2_ electroreduction to CO on Fe‐N_4_ moieties. d) First shell fitting of Fourier transformation of EXAFS spectra for NiPor‐CTF and the model NiN_4_ structure in NiPor‐CTF. e) The first derivative of the Fe‐edge XANES spectra at different potentials. a) Reproduced with permission.^[^ ^10^ ^]^ Copyright 2017, American Chemical Society. b) Reproduced with permission.^[^ ^62^ ^]^ Copyright 2018, Springer Nature. c) Adapted with permission.^[^ ^81^ ^]^ Copyright 2018, WILEY‐VCH. d) Reproduced with permission.^[^ ^61^ ^]^ Copyright 2019, WILEY‐VCH. e) Adapted with permission.^[^ ^46^ ^]^ Copyright 2019, AAAS.

Similar to homogeneous M‐cyclam^[^ ^42^ ^]^ materials, M‐N_4_ moieties in heterogeneous M‐N‐C electrocatalysts were widely considered as the active sites for CO_2_ electroreduction to CO.^[^ ^47^, ^54^, ^56^ ^]^ For example, Wu et al.^[^ ^10^ ^]^ established a pyridine‐type Ni‐N_4_ structure via a topo‐chemical transformation method, which could avoid agglomeration of nickel species and ensure maximum maintenance of the Ni‐N_4_ structure (**Figure** **6a**). The obtained Ni‐N_4_‐C catalyst with abundant active sites exhibited a maximum CO selectivity up to 99% with a high *j*_CO_ up to 28.3 mA cm^−2^ at ‐0.81 V. DFT calculation results showed that the high CO_2_RR activity could be attributed to the reduced binding energy of COOH* by pyridine‐type Ni‐N_4_ (1.7 eV) sites. However, it should be noted that the calculated *COOH formation energy of 1.7 eV is still a bit too high, higher than Δ*G*_H_,^[^ ^10^, ^47^, ^55^ ^]^ which is contradictory to the experimental observation of high CO selectivity over H_2_. Liu and co‐workers proved atomically dispersed low‐valent Ni(I) as the active site for CO_2_ electroreduction by performing operando XAS measurements.^[^ ^62^ ^]^ During electrolysis, the unpaired electron in the Ni 3d_x2−y2_ orbital would be delocalized and the charge would spontaneously mitigate from Ni(I) to the carbon 2p orbital in CO_2_ to generate a CO_2_^*δ*−^ species (Figure 6b). The obtained SAC maintained a superior CO FE up to 98% with a *j*_CO_ of 22 mA cm^−2^ for 100 h at ‐0.72 V.

Pyridine‐type Fe‐N_4_ sites have been shown to bind CO strongly, leading to limited CO selectivity in CO_2_RR. Pyridine‐type Fe‐N_4_ catalysts with different graphitic nitrogen doping configurations were further studied (Figure 6c).^[^ ^81^ ^]^ The N atoms substitution on graphene was found able to reduce the energy barrier for COOH* formation over FeN_4_ sites (from 0.63 to 0.29 eV), and thus facilitated CO* desorption (from 0.5 to 0.3 eV).

In addition to the pyridine‐type M‐N_4_ sites, pyrrole‐type M‐N_4_ sites can also reduce CO_2_ to CO. For instance, a Ni porphyrin‐based covalent triazine framework (NiPor‐CTF) catalyst with abundant atomically dispersed pyrrole‐type NiN_4_ sites (Figure 6d) was prepared through a conventional ionothermal strategy for effective electrochemical CO_2_ reduction.^[^ ^61^ ^]^ The NiPor‐CTF achieved a high CO productivity with a maximum CO FE up to 97% and a *j*_CO_ of 51.3 mA cm^−2^ at −0.9 V versus RHE for 20 h. Theoretical calculations indicated that the Gibbs free energy of *COOH on pyrrole‐type NiN_4_ sites (1.49 eV) embedded in CTF skeleton slightly reduced as compared to that on pyridine‐type NiN_4_ (1.55 eV), explaining the improved CO_2_RR performance as compared to Ni/N‐PC with pyridine‐type NiN_4_. Moreover, the type of N ligands could affect the oxidation state of the central metal. Fe^3+^ atoms coordinated with pyrrolic N (Fe^2+^‐N‐C) exhibited a high CO FE of over 80% in CO_2_RR in a low potential range of ‐0.2– ‐0.5 V versus RHE, reaching a *j*_CO_ of 20 mA cm^−2^ at ‐0.47 V, better than Fe^2+^ atoms coordinated with pyridinic N (Fe^2+^‐N‐C).^[^ ^46^ ^]^ This result was attributed to the pyrrolic N ligands, which enabled stabilizing Fe^3+^ during electrocatalysis, promoting CO_2_ adsorption and CO desorption. If the applied potential was decreased to less than ‐0.5 V versus RHE, Fe^3+^ sites would be reduced to Fe^2+^ with coordination number decreased from 4 to 3 (Figure 6e), leading to a significant decrease in CO_2_RR activity.

#### 3.2.2 M‐N_3_ Moieties

**Figure 7:**
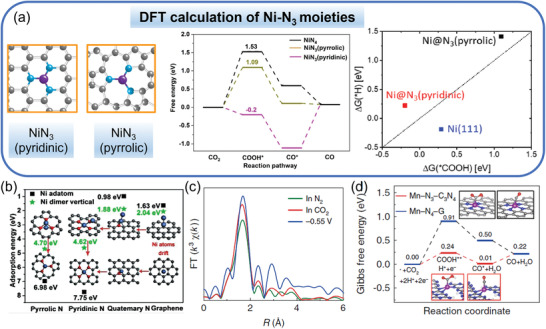
M‐N‐C catalysts with MN_3_ sites for CO_2_‐to‐CO conversion. a) Optimized atomic structures including NiN_3_, NiN_3_V, and NiN_2_V_2_ as well as DFT calculated free energy profiles for CO_2_ electroreduction and comparison of Δ*G*(*H) and Δ*G*(*COOH). C, N, and Ni atoms are represented by gray, blue, and purple spheres, respectively. b) The adsorption energies of Ni adatom and Ni dimer vertical of pyrrolic N, pyridinic N, quaternary N, and graphene. All values are in eV per Ni atom. c) EXAFS spectra at the Mn K‐edge of Mn–C_3_N_4_/CNT under various conditions. d) Calculated Gibbs free energy diagrams for CO_2_RR on Mn‐N_3_‐C_3_N_4_ and Mn‐N_4_‐G. a) Reproduced with permission.^[^ ^75^ ^]^ Copyright 2019, WILEY‐VCH. b) Adapted with permission.^[^ ^76^ ^]^ Copyright 2018, WILEY‐VCH. c,d) Adapted with permission.^[^ ^82^ ^]^ Copyright 2020, Springer Nature.

Although it has been reported that HER dominates on the NiN_3_ site (pyridinic),^[^ ^73^ ^]^ there are a few studies predicting that the Ni@N_3_ (pyrrolic) sites are likely the major active sites for CO_2_ electroreduction.^[^ ^75^, ^76^ ^]^ Single Ni atom catalyst with a possible NiN_3_ structure on N‐doped carbon nanotubes (named as NC‐CNTs (Ni)) was prepared through an in situ thermal transformation method.^[^ ^75^ ^]^ The obtained NC‐CNTs (Ni) possessed NiN_3_ moieties, proved by extended X‐ray absorption fine structure (EXAFS), and showed a CO FE over 90% as well as a *j*_CO_ of ≈10 mA cm^−2^ at −1.0 V versus RHE. Importantly, this study provided a DFT comparison of the two different Ni@N_3_ sites including Ni@N_3_(pyridinic) and Ni@N_3_(pyrrolic) site for CO_2_‐to‐CO reduction (**Figure** **7a**). The calculations revealed that the binding energy of *COOH on the Ni@N_3_(pyrrolic) site (1.09 eV) was lower than that on the Ni@N_4_ site (1.54 eV), implying that Ni@N_3_(pyrrolic) site was more active for CO_2_ reduction to CO than the Ni@N_4_ site. Besides, the desorption of CO on the Ni@N_3_(pyrrolic) site was exothermic, suggesting less *CO poisoning. Moreover, Δ*G*(*COOH) was more negative than Δ*G*(*H) on both pyridinic and pyrrolic Ni@N_3_ moieties, illustrating that CO_2_RR was thermodynamically more preferred than HER. Optimization of Ni atom structures on pure and N‐doped graphene was performed via DFT by Wu et al.,^[^ ^76^ ^]^ which revealed that the most stable adsorption sites were pyrrolic N (binding energy 6.98 eV) and pyridinic N (binding energy 7.75 eV) (Figure 7b). Therefore, three structures including Ni‐4N and Ni‐3N (Ni@N_3_(pyrrolic)) representing Ni SAs and Ni (111) standing for Ni NPs were comparatively studied, which indicated that Ni‐3N (Ni@N_3_(pyrrolic)) was more active than Ni‐4N in CO_2_RR to CO due to its lower *COOH binding energy while Ni NPs were inactive due to its high required energy for CO desorption. Apart from Ni‐N‐C, Mn‐N‐C with Mn‐N_3_ sites embedded in g‐C_3_N_4_ on carbon nanotubes (Mn‐C_3_N_4_/CNT) was also constructed, which exhibited a CO selectivity up to 98.8% with a *j*_CO_ of 14.0 mA cm^−2^ at −0.55 V versus RHE, outperforming all of the Mn‐N‐C electrocatalysts previously reported.^[^ ^82^ ^]^ In situ XAS analysis as well as DFT calculations revealed that the outstanding performance was derived from the Mn‐N_3_ sites that facilitated the COOH* intermediate formation by decreasing the free energy barrier compared to Mn‐N_4_ sites (Figure 7c,d).

#### 3.2.3 M‐N_5‐6_ moieties

**Figure 8:**
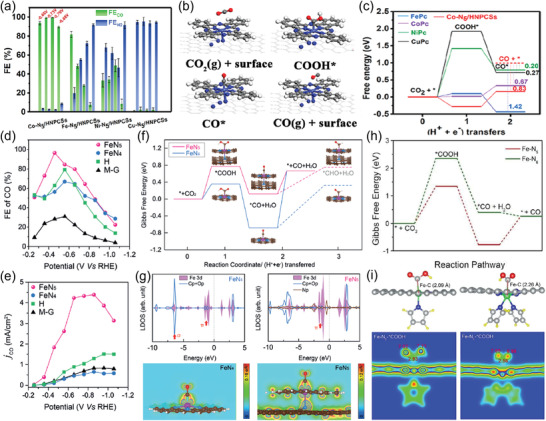
M‐N‐C with M‐N_5_ structure for CO_2_‐to‐CO conversion. a) CO FEs and H_2_ FEs of M‐N_5_/HNPCSs (M = Co, Fe, Ni, Cu). b) Optimized structures for the intermediates. c) Calculated free energy diagrams for CO_2_ electroreduction to CO. d) CO FEs versus different applied potentials. e) *j*_CO_ versus different applied potentials. f) Calculated Gibbs free energy diagrams for CO_2_ electroreduction to CO on Fe‐N_4_ and Fe‐N_5_. g) Local DOS and partial charge density of the FeN_4_ and FeN_5_ with adsorbed CO. h) Calculated free energy diagrams for CO_2_ electroreduction to CO on Fe‐N_5_ and Fe‐N_6_. i) Optimized structures of Fe‐N_5_ and Fe‐N_6_ and a slice of calculated charge densities with adsorbed *COOH. a–c) Adapted with permission.^[^ ^84^ ^]^ Copyright 2018, American Chemical Society. d–g) Adapted with permission.^[^ ^83^ ^]^ Copyright 2019, Wiley‐VCH. h,i) Adapted with permission.^[^ ^85^ ^]^ Copyright 2020, The Royal Society of Chemistry.

M‐N‐C electrocatalysts with the central metal atom M coordinated with more than four N atoms were also recently studied. For instance, Co‐N‐C and Fe‐N‐C with atomically dispersed CoN_5_ and FeN_5_ sites, respectively were both reported to be extremely efficient in CO_2_‐to‐CO conversion.^[^ ^83^, ^84^ ^]^ Pan et al.^[^ ^84^ ^]^ synthesized a class of M‐N_5_/HNPCSs (M = Co, Fe, Ni, Cu) catalysts with dominant atomically dispersed M−N_5_ sites and compared their performance for CO_2_ to CO production. Co‐N_5_/HNPCSs exhibited the highest CO FE of 99.4% at −0.79 V versus RHE (**Figure** **8a**), outperforming most other previously reported Co‐N‐C electrocatalysts. The Co‐N_5_ moieties were determined to be the active sites for CO_2_RR, which promoted COOH* formation as well as CO desorption (Figure 8b‐c). The Fe‐N_5_ catalyst with superior CO FE and *j*_CO_ to the Fe‐N_4_ catalyst was also reported by Zhang et al. (Figure 8d,e).^[^ ^83^ ^]^ Theoretical calculations (Figure 8f) show that the rate‐determining step on FeN_4_ is the *CO desorption that requires an energy of 1.35 eV, while that over FeN_5_ is the *COOH formation step with an energy barrier of 0.77 eV. Moreover, as shown in Figure 8g, the axial pyrrolic‐N ligand on the FeN_5_ sites could deplete the electron density of Fe 3d orbitals and reduce the Fe‐CO p back‐donation as compared to FeN_4_, hence leading to fast desorption of CO. Furthermore, Fe‐N_6_ catalyst was compared to Fe‐N_5_ by Chen and co‐workers,^[^ ^85^ ^]^ in which, Fe‐N_5_ catalyst reached a lower overpotential (50 mV) and exhibited a higher CO FE (up to 99%), *j*_CO_ and CO TOF than the Fe‐N_6_ catalyst in a broad potential range of −0.35–−1.05 V versus RHE. The superior performance was attributed to the Fe‐N_5_ site, which facilitated the *COOH formation (Figure 8h,i).

#### 3.2.4 M‐N*_x_* C_4‐_ *_x_* (*x* = 1–3) Moieties

**Figure 9:**
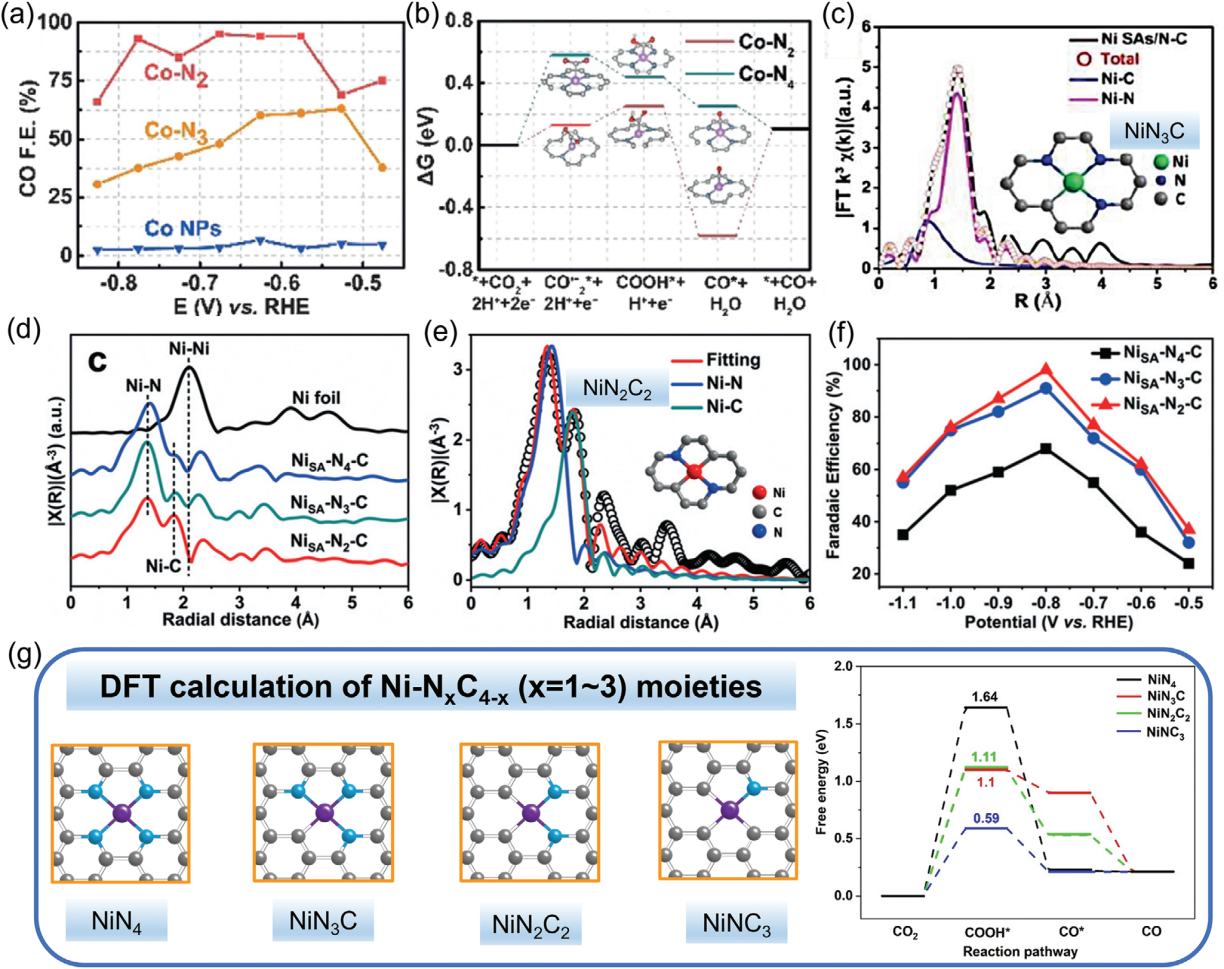
M‐N‐C catalysts with different MN*_x_* C_4‐_ *_x_* sites for CO_2_‐to‐CO conversion. a) CO FE versus applied potential for Co‐N_2_, Co‐N_3,_ and Co NPs catalysts. b) Calculated Gibbs free energy diagrams for CO_2_ electroreduction to CO on Co‐N_2_ and Co‐N_4_. c) EXAFS fitting curves for Ni SAs/N‐C showing NiN_3_C structure. d,e) EXAFS and fitting curves for Ni_SA_‐N_2_‐C showing NiN_2_C_2_ structure f) CO FE versus applied potential for Ni_SA_‐N*_X_*‐C catalyst. g) Optimized atomic structures including NiN_4_, NiN_3_C, NiN_2_C_2_, and NiNC_3_ as well as DFT calculated free energy profiles for CO_2_ electroreduction. C, N, and Ni atoms are represented by gray, blue, and purple spheres, respectively. a,b) Adapted with permission.^[^ ^86^ ^]^ Copyright 2018, Wiley‐VCH. c) Reproduced with permission.^[^ ^71^ ^]^ Copyright 2017, American Chemical Society. d–f) Adapted with permission.^[^ ^78^ ^]^ Copyright 2020, Wiley‐VCH. g) Reproduced with permission.^[^ ^87^ ^]^ Copyright 2019, Elsevier Inc.

In addition to the typical M‐N_4_ structure, the M‐N and M‐C mixed structures were also studied for electrochemical CO_2_ reduction to CO. Wu et al.^[^ ^86^ ^]^ prepared atomically dispersed Co on N‐doped carbon with different M‐N/C mixed structures (Co‐N_4_, Co‐N_3_C, or Co‐N_2_C_2_) by changing pyrolysis temperature. It was found that the Co‐N_2_ (Co‐N_2_C_2_) catalyst reached a CO selectivity up to 94% with a total current density of 18.1 mA cm^−2^ at ‐0.63 V versus RHE, far superior to that for Co‐N_3_ and Co‐N_4_ (**Figure** **9a**). DFT results (Figure 9b) indicated that the low coordination number of Co‐N_2_ could facilitate CO_2_ activation and thus lead to enhanced CO_2_RR activity. However, the strong binding of CO* on the Co‐N_2_ site still inhibited CO formation. On the contrary, some works reported that Co‐N_4_ sites owned superior performance to Co‐N_4‐_ *_x_* C*_x_* sites.^[^ ^45^ ^]^

Additionally, a Ni single‐atom catalyst (SAC) with a dominant NiN_3_C structure was prepared by an ionic exchange process.^[^ ^71^ ^]^ The obtained Ni single‐atom catalyst (Ni SAs/N‐C), in which the Ni atoms were anchored with three N atoms and one C atom in the carbon matrix according to the XAFs fitting (Figure 9c), was able to reduce CO_2_ to CO with a selectivity of ≈71.9% at ‐1.0 V. The NiN_3_C structure was considered as the active site that exhibited strong bonding to CO_2_^•−^. In another work, NiN_2_C_2_ structure was reported more active in CO_2_RR than NiN_3_C and NiN_4_.^[^ ^78^ ^]^ A series of Ni_SA_‐N*_x_*‐C catalysts (*x* = 2–4) with different N coordination numbers (Figure 9d,e) were prepared by changing the pyrolysis temperature and studied for CO_2_RR. The Ni_SA_‐N_2_‐C catalyst with the lowest N coordination number suggested the most superior performance for CO production with CO FE up to 98% (Figure 9f). DFT calculations revealed that the low N coordination number was favorable to COOH* intermediate formation. Similar DFT calculations for different NiN*_x_* C_4‐_ *_x_* structures including NiN_4_, NiN_3_C, NiN_2_C_2_, NiNC_3_ for CO_2_‐to‐CO electroreduction were performed in other works.^[^ ^78^, ^87^, ^88^ ^]^ As shown in Figure 9g, the results suggested that NiNC_3_ (0.59 eV) was the most favorable site for COOH* formation, followed by NiN_3_C (1.10 eV), NiN_2_C_2_ (1.11 eV), and NiN_4_ (1.64 eV). Besides, for these different NiN_x_ structures, CO* desorption step is exothermic, revealing easy desorption of adsorbed CO* intermediate to release molecular CO.

Furthermore, the CO_2_RR performance of some M‐N‐C catalysts with these four structures (typical M‐N_4_ structure, M‐N_3_ structure, M‐N_5∼6_ structure and M‐N*_x_* C_4‐_ *_x_* (*x* = 1–3) structures) was compared as shown in **Table** **1**. For the CO_2_RR performance comparison of M‐N‐C catalysts with different metal centers, the nature of the metal centers is still the decisive factor. While for catalysts with the same metal center, the coordination structure has different effects. Notably, most of the coordination structures reported in the literature were only an average structure determined by EXAFS, not a homogeneous coordination structure. Therefore, it is necessary to further develop M‐N‐C catalysts with a homogeneous coordination structure to accurately compare the activity of different coordinative active sites

**Table 1:** Comparison of CO_2_RR performance for M‐N‐C catalysts with four structures (typical M‐N_4_ structure, M‐N_3_ structure, M‐N_5∼6_ structure and M‐N*_x_* C_4‐_ *_x_* (*x* = 1–3) structures)

M‐N‐C catalyst	Active site(s)	Metal content(wt%)	Onset potential (vs RHE)	CO FE[%]	*j*_CO_[mA cm^−2^]	TOF[h^−1^]
Ni‐N_4_‐C^[^ ^10^ ^]^	Pyridine‐type Ni‐N_4_	1.41	−0.4 V	99 @−0.81 V (vs RHE)	28.3 @−0.81 V (vs RHE)	/
A‐Ni‐NG^[^ ^62^ ^]^	Pyridine‐type Ni‐N_4_	4.6	−0.35 V	97 @−0.72 V (vs RHE)	30.6 @−0.72 V (vs RHE)	8000 @−0.72 V (vs RHE)
A‐Ni‐NSG^[^ ^62^ ^]^	Pyridine‐type Ni‐N_4_	2.5	−0.25 V	97 @−0.72 V (vs RHE)	22 @−0.72 V (vs RHE)	14 800 @−0.72 V (vs RHE)
Fe/NG‐750^[^ ^81^ ^]^	Pyridine‐type FeN_4_	1.25	−0.3 V	80 @−0.57 V (vs RHE)	2 @−0.6 V (vs RHE)	/
NiPor‐CTF^[^ ^61^ ^]^	Pyrrole‐type Ni‐N_4_	2.4	−0.44 V	97 @−0.9 V (vs RHE)	51.3 @−0.9 V (vs RHE)	1690 @−0.9 V (vs RHE)
NC‐CNTs (Ni)^[^ ^75^ ^]^	NiN_3_	1.01	−0.4 V	90 @−0.8 V (vs RHE)	9.2 @−1 V (vs RHE)	11 650 @−0.89 V (vs RHE)
SE‐Ni SAs@PNC^[^ ^76^ ^]^	NiN_3_ NiN_4_	/	/	95.7 @−0.8 V (vs RHE)	18.3 @−1 V (vs RHE)	47 805 @−1 V (vs RHE)
Mn‐C_3_N_4_/CNT^[^ ^82^ ^]^	Mn‐N_3_	0.17	−0.3 V	98.8 @−0.55 V (vs RHE)	14 @−0.55 V (vs RHE)	/
Ni‐N_5_/HNPCSs^[^ ^84^ ^]^	NiN_5_	3.32	/	50 @−0.79 V (vs RHE)	/	/
Fe‐N_5_/HNPCSs^[^ ^84^ ^]^	FeN_5_	3.03	/	82 @−0.66 V (vs RHE)	/	/
Co‐N_5_/HNPCSs^[^ ^84^ ^]^	CoN_5_	3.54	/	99.4 @−0.79 V (vs RHE)	4.5 @−0.73 V (vs RHE)	480.2 @−0.73 V (vs RHE)
Cu‐N_5_/HNPCSs^[^ ^84^ ^]^	CuN_5_	3.75	/	2 @−0.73 V (vs. RHE)	/	/
Fe‐N_5_^[^ ^83^ ^]^	FeN_5_	0.8 at%	−0.26 V	97 @−0.46 V (vs RHE)	1.81 @−0.46 V (vs RHE)	/
Fe‐N_4_^[^ ^83^ ^]^	FeN_4_	/	/	66 @−0.56 V (vs RHE)	0.3 @−0.56 V (vs RHE)	/
Fe‐N_5_^[^ ^85^ ^]^	FeN_5_	0.57	−0.16 V	99 @−0.35 V (vs RHE)	20.8 @−1.05 V (vs RHE)	5006 @−1.05 V (vs RHE)
Fe‐N_6_^[^ ^85^ ^]^	FeN_6_	0.58	−0.2 V	96 @−0.35 V (vs RHE)	5.5 @−1.05 V (vs RHE)	1324 @−1.05 V (vs RHE)
Ni SAs/N‐C^[^ ^71^ ^]^	NiN_3_C	1.53	−0.57 V	71.9 @−0.9 V (vs RHE)	7.37 @−1 V (vs RHE)	5273 @−1 V (vs RHE)
Co‐N_2_^[^ ^86^ ^]^	CoN_2_C_2_	0.25	−0.22 V	94 @−0.63 V (vs RHE)	17 @−0.63 V (vs RHE)	18 200 @−0.63 V (vs RHE)
Co‐N_3_^[^ ^86^ ^]^	CoN_3_C	/	−0.45 V	63 @−0.53 V (vs RHE)	1.56 @−0.53 V (vs RHE)	1250 @−0.63 V (vs RHE)
Co‐N_4_^[^ ^86^ ^]^	Pyridine‐type CoN_4_	/	/	4.2 @−0.83 V (vs RHE)	/	84 @−0.83 V (vs RHE)
Co_1_‐N_4_^[^ ^45^ ^]^	Pyridine‐type Co‐N_4_	0.6	−0.3 V	82 @−0.8 V (vsRHE)	15.8 @−0.8 V (vs RHE)	1455 @−1.0 V (vs RHE)
Co_1_‐N_4‐_ *_x_* ^[^ ^45^ ^]^	Co‐N*_x_*	0.63	−0.32 V	47 @−0.8 V (vs RHE)	8.8 @−0.8 V (vs RHE)	763 @−1.0 V (vs RHE)

### 3.3 Engineering the Second and Higher Shell of the Metal Coordination Environment

#### 3.3.1 Vacancy Engineering

**Figure 10:**
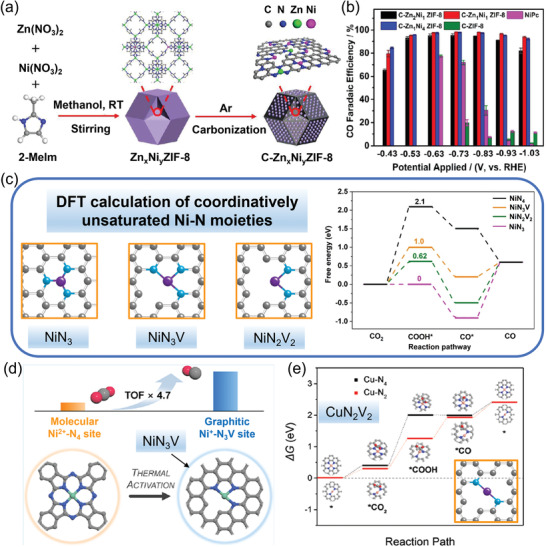
M‐N‐C catalysts with coordinatively unsaturated M‐N structures for CO_2_‐to‐CO conversion. a) Scheme of C‐Zn*_x_* Ni*_y_* ZIF‐8 synthesis. b) CO FE versus applied potential for various C‐Zn*_x_* Ni*_y_* ZIF‐8 catalysts. c) Optimized atomic structures including NiN_3_, NiN_3_V, and NiN_2_V_2_ as well as DFT calculated free energy profiles for CO_2_RR. C, N, and Ni atoms are represented by gray, blue, and purple spheres, respectively. d) Illustration showing higher CO_2_‐to‐CO TOFs of H‐NiPc/CNT with NiN_3_V sites than NiPc/CNT with NiN_4_ sites. e) Calculated Gibbs free energy diagrams for CO_2_ electroreduction to CO on Cu‐N_2_ and Cu‐N_4_. a–c) Reproduced with permission.^[^ ^73^ ^]^ Copyright 2018, The Royal Society of Chemistry. d) Reproduced with permission.^[^ ^68^ ^]^ Copyright 2020, American Chemical Society. e) Reproduced with permission.^[^ ^91^ ^]^ Copyright 2019, WILEY‐VCH.

Some studies found that coordinatively unsaturated M‐N sites formed by engineering surface vacancies in carbon matrix could improve the binding strength of reaction intermediates and thus reduce the reaction free energy of CO_2_RR.^[^ ^73^, ^74^, ^89^, ^90^ ^]^ For instance, a series of improved CO_2_RR electrocatalysts derived from Zn/Ni bimetallic ZIF‐8 with different proportions of Zn and Ni were synthesized (**Figure** **10a**), in which coordinatively unsaturated Ni‐N sites were embodied in porous carbon matrix.^[^ ^73^ ^]^ In this work, four Ni‐N architectures were compared by DFT, including NiN_4_, NiN_3_, NiN_3_V, and NiN_2_V_2_, where V represents coordination vacancy (Figure 10c). According to the calculation results, the higher activity of CO_2_RR (Figure 10b) could be attributed to the significantly lower free energy of *COOH on the coordinatively unsaturated Ni‐N sites as compared to that on NiN_4_. Similar calculation results were also reported in other recent studies.^[^ ^74^, ^80^ ^]^ Besides, Sa et al.^[^ ^68^ ^]^ and Lu et al.^[^ ^79^ ^]^ prepared vacancy‐defect Ni SAC to compare with typical NiN_4_ SAC, and found that the vacancy‐defect Ni‐N‐C with dominant NiN_3_V sites established high CO turnover frequency (13860 h^−1^ at −0.94 V vs RHE and 135 000 h^−1^ at −0.9 V vs RHE, respectively) for CO_2_ reduction to CO, far superior to the catalysts with NiN_4_ sites (Figure 10d). Additionally, a novel Cu‐N_2_/GN catalyst with atomically dispersed undercoordinated Cu‐N_2_V_2_ sites on graphene matrix was reported, which exhibited high electrocatalytic activity and selectivity (a maximum CO FE of 81%) for CO_2_RR, showing an onset potential of −0.33 V versus RHE. ^[^ ^91^ ^]^ According to DFT calculation, the shorter bonding length of the Cu‐N_2_ site as compared to that of the Cu–N_4_ site boosted the *COOH and then the *CO formation by accelerating electron transfer from Cu‐N_2_ site to *CO_2_ (Figure 10e).

#### 3.3.2 Edge‐Anchored Structure

**Figure 11:**
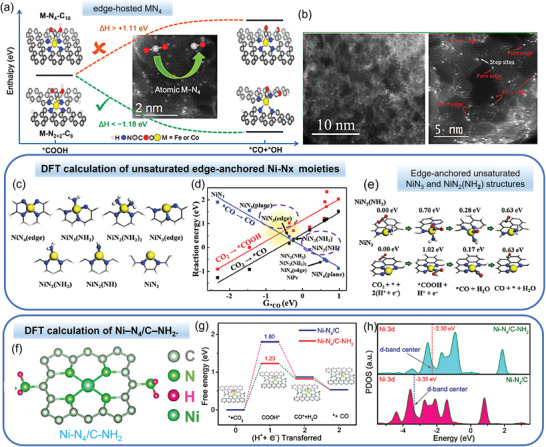
M‐N‐C catalysts with edge‐anchored Ni‐N*_x_* structures or with amino modification for CO_2_‐to‐CO conversion. a) The initial and final states for the *COOH dissociation reaction on M‐N_4_‐C_10_ and M‐N_2+2_‐C_8_ sites (M = Fe, Co). b) AC‐STEM images showing that the single‐atom Ni sites are predominately anchored at the edges of nanopores inside Ni‐N‐MEGO. DFT calculation results: c) Different Ni‐N active site structures on the edges of graphene sheets. d) The reaction energy is in a linear relationship with the adsorption free energy of *CO (G*CO) on the active sites. Lower reaction energy indicates higher reactivity. A lower G*CO indicates weaker adsorption. e) Reaction pathway on the NiN_2_(NH_2_) and NiN_3_ site, with the free energy shown on top. (C: gray; N: blue; Ni: yellow; O: red; and H: white). f) Schematic of the local coordination environment for Ni‐N_4_/C‐NH_2_. g) Calculated Gibbs free energy diagrams for CO_2_ electroreduction to CO on Ni‐N_4_/C‐NH_2_ and Ni‐N_4_/C. h) Projected DOS of Ni 3d in Ni‐N_4_/C‐NH_2_ and Ni‐N_4_/C. a) Adapted with permission.^[^ ^92^ ^]^ Copyright 2018, American Chemical Society. b–e) Reproduced with permission.^[^ ^77^ ^]^ Copyright 2019, Elsevier B.V. f–h) Reproduced with permission.^[^ ^69^ ^]^ Copyright 2021, The Royal Society of Chemistry.

It should be noted that the above studies only paid attention to the bulk M‐N_4_ sites for the CO_2_RR while neglecting the active sites at the edges. In this regard, Wang's group prepared M‐N‐C (M = Fe, Co) catalysts containing both bulk and edge M−N_4_ sites by employing Fe‐ or Co‐doped MOF as precursors.^[^ ^92^ ^]^ The electrocatalytic activity of bulk and edge M‐N_4_ sites were compared in DFT calculations (**Figure** **11a**), which found that the COOH dissociation to CO* and OH* was endothermic on M‐N_4_‐C_10_ (>1.11 eV) while it was exothermic on M‐N_2+2_‐C_8_ (←1.18 eV). Therefore, the edge M‐N_2+2_ site was found more active in catalyzing CO_2_RR than the bulk M‐N_4_ site. The same conclusion was also reached in the Ni‐N‐C catalyst system.^[^ ^63^ ^]^

In addition, unsaturated edge‐anchored metal single atoms for electrochemical CO_2_ reduction were also studied in comparison to the unsaturated in‐plane M‐N structures. Jiang et al.^[^ ^77^ ^]^ used microwave exfoliated GO as the porous support to prepare single Ni atom catalysts (Ni‐N‐MEGO), generating mangy edge‐anchored single Ni atoms distributed along the edges of nanopores (<6 nm) (Figure 11b). The Ni‐N‐MEGO catalyst achieved a high CO selectivity of 92.1% at the potential of ‐0.7 V versus RHE in CO_2_RR. Several different possible Ni‐N*_x_* (*x* = 2–4) structures were compared (Figure 11c) and the reaction energies were computed to assess CO_2_RR activity (Figure 11d). As shown in Figure 11e, it could be deduced that edge‐coordinated unsaturated NiN_3_ and NiN_2_(NH_2_) architectures possessed the highest activity because of their optimized energies for both CO_2_ activation (0.7 and 1.02 eV) and CO desorption (0.63 and 0.63 eV), while NiN_2_ was inactive because of the high CO desorption energy.

#### 3.3.3 Amino Modification

As amino‐modified carbon materials showed enhanced CO_2_ adsorption capacity, Liu's group^[^ ^69^ ^]^ modified the M‐N‐C electrocatalysts with amino functional groups with the well‐maintained atomically dispersed structure to improve the CO_2_RR activity (Figure 11f). The obtained Ni‐N_4_/C‐NH_2_ catalyst reached CO selectivities over 90% in a broad potential range, and achieved a *j*_CO_ up to 63.6 mA cm^−2^ at ‐1.0 V versus RHE in a H‐type cell, 2.5 times that of Ni‐N_4_/C catalyst. Moreover, an industrial level current density up to 440 mA cm^−2^ with a relatively high CO selectivity over 85% was achieved in a flow cell. The superior performance was due to the reduced free energy of the COOH* intermediate derived from the regulation of the electronic distribution by amino modification (Figure 11g). The more positive d‐band center of the Ni 3d DOS in Ni‐N_4_/C‐NH_2_ than that in Ni‐N_4_/C benefited the adsorption strength of the reaction intermediates due to declined occupation (Figure 11h).

### 3.4 Other Heteroatom Doping

**Figure 12:**
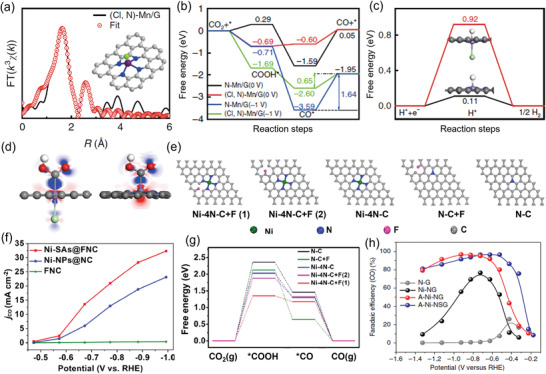
M‐N‐C catalysts with other heteroatoms doping for CO_2_‐to‐CO conversion. a) EXAFS fitting curves of the (Cl, N)‐Mn/G in R space. Inset shows the schematic model of (Cl, N)‐Mn/G. b) Calculated free energy of CO_2_RR. c) Calculated free energy of hydrogen adsorption. d) Electron density difference for COOH* adsorbed on (Cl, N)‐Mn/G (left) and N‐Mn/G (right). The blue and red region denote the electron accumulation and electron depletion, respectively. e) Different model structures of the F‐tuned single‐Ni‐atom catalyst. f) *j*_CO_ versus applied potential. g) Calculated Gibbs free energy diagrams for CO_2_‐to‐CO conversion. h) CO Faradaic efficiency at various applied potentials. a–d) Adapted with permission.^[^ ^97^ ^]^ Copyright 2019, Springer Nature. e–g) Adapted with permission.^[^ ^70^ ^]^ Copyright 2021, Elsevier B.V. h) Adapted with permission.^[^ ^62^ ^]^ Copyright 2018, Springer Nature.

The introduction of other heteroatoms (such as B, O, F, P, S, and Cl) into N‐doped carbon to tune the electronic structure of single metal atoms provides another effective method to enhance electrocatalytic activity of the reaction.^[^ ^93^, ^94^, ^95^, ^96^ ^]^ This strategy can manipulate the first shell of the coordination environment adjacent to the central metal atom or the higher shell bonded to the atom of the first shell to tune the electronic structure. For instance, although Mn‐N‐C catalysts with dominant MnN_4_ sites showed low CO selectivity in CO_2_RR, halogen (Cl/F/Br) and nitrogen dual‐coordinated single Mn atom catalysts were found efficient in reducing CO_2_ to CO.^[^ ^97^ ^]^ The single Mn atom in (Cl, N)‐Mn/G was determined to be coordinated with a Cl atom in the axial direction and four N atoms in plane by EXAFS fitting analysis (**Figure** **12a**). DFT calculation suggested obviously decreased free energy barrier for the rate‐limiting step of CO desorption (Figure 12b), at the same time inhibiting HER (Figure 12c), which was because of the modified electronic structure of the single Mn atom active site via electron transfer between Mn and Cl (Figure 12d). A Ni‐based single‐atom catalyst (Ni‐N_4_‐O/C) with a similar coordination structure, in which atomically dispersed Ni sites were coordinated with an O atom in the axial direction and four N atoms in plane, also exhibited outstanding CO_2_RR performance with a maximum CO selectivity up to 100%.^[^ ^98^ ^]^ Besides, heteroatom doping into carbon matrix beyond the first shell of the central metal also has an impact on the distribution of electron density around the central metal atom, leading to changes in catalytic activity. For example, a F‐tuned single‐Ni‐atom catalyst with NiN_4_ structure (Figure 12e) was found to be capable of reducing CO_2_ to CO in a higher efficiency than its counterpart without F‐doping (Figure 12f), which was attributed to the lower energy barrier for CO_2_ activation (Figure 12g).^[^ ^70^ ^]^ N, S co‐doped Ni SAC showed a reduced overpotential compared to N‐doped Ni SAC catalyst (Figure 12h).^[^ ^62^ ^]^

### 3.5 Dual‐Atom Site Catalysts

Dual‐atom site catalysts are emerging as a promising candidate for electrochemical reactions.^[^ ^99^, ^100^, ^101^, ^102^ ^]^ According to different relative positions of dual‐atom sites, they can be classified into isolated dual‐atom site catalysts and binuclear dual‐atom site catalysts.

**Figure 13:**
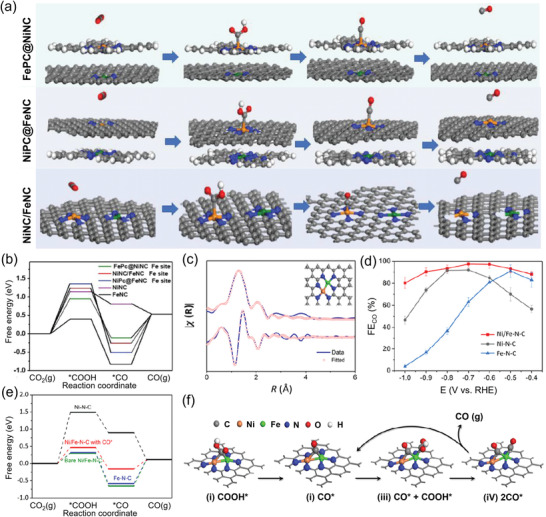
M_1_M_2_‐N‐C catalysts with dual‐atom sites for CO_2_‐to‐CO conversion. a) Schematic atomic structure of CO_2_RR on the Fe site in FePc@NiNC, NiPc@FeNC, and NiNC/FeNC. b) Calculated free energy of CO_2_RR. c) Fourier transformation of the EXAFS spectra at R space of Ni/Fe‐N‐C. d) CO FE versus applied potential of Ni/Fe‐N‐C, Ni‐N‐C, Fe‐N‐C catalysts. e) Calculated free energy of CO_2_RR. f) The catalytic mechanism on diatomic metal‐nitrogen site based on the optimized structures of adsorbed intermediates COOH* and CO*. a,b) Adapted with permission.^[^ ^103^ ^]^ Copyright 2021, Elsevier Ltd. c–f) Adapted with permission.^[^ ^101^ ^]^ Copyright 2019, WILEY‐VCH.

Isolated dual‐atom site denotes two types of isolated central metal atoms anchored with surrounding chelating atoms, without forming metal–metal bond, which have been reported for CO_2_RR to generate syngas (CO/H_2_). For example, Chen and co‐workers reported a series of NiFe dual‐atom catalysts containing bimetallic centers for tunable syngas production (from 1:3 to 4:1) by changing the configurations of the metal‐N sites (Ni‐N_4_ and Fe‐N_4_), as well as tuning the applied potential.^[^ ^103^ ^]^ Theoretical and experimental results revealed that there was a synergy of Ni‐N_4_ and Fe‐N_4_ sites for CO_2_RR process, where the Fe atoms functioned both as the reactive and adsorption sites of intermediates for CO_2_‐to‐CO conversion, while the introduction of Ni atoms lowered the bond strength of Fe atoms to CO* (**Figure** **13a,b**). Moreover, a CoNi bimetallic atom catalyst with isolated CoN_4_ and NiN_4_ moieties was developed, which showed a high total current density > 74 mA cm^−2^ with CO/H_2_ ratios tunable from 0.23 to 2.26 in CO_2_RR.^[^ ^104^ ^]^

In addition to isolated dual‐atom sites, binuclear dual‐atom sites can also be formed during catalyst preparation, for which the two central metal atoms are bonded to each other to form dual‐metal atom pairs. Recently, a dual‐metal‐atom electrocatalyst comprising isolated diatomic Ni‐Fe sites anchored on N‐doped carbon (Figure 13c) was reported for CO_2_ electroreduction.^[^ ^101^ ^]^ As shown in Figure 13d, the NiFe dual‐atom catalyst (Ni/Fe‐N‐C) outperformed Fe‐N‐C and Ni‐N‐C in the potential range of ‐0.4– ‐1.0 V versus RHE, achieving a maximum CO FE up to 98% at ‐0.7 V. Theoretical results revealed that although bare Ni/Fe‐N‐C tended to be passivated by strongly bounded CO* intermediate, the energy barrier for the rate‐determining step on CO‐adsorbed Ni/Fe‐N‐C was significantly reduced in contrast with to bare Ni/Fe‐N‐C (Figure 13e). Based on which, the CO_2_RR mechanism on the dual‐metal‐atom site was proposed (Figure 13f) that the neighboring NiFe dual‐site was first passivated by strong bonding to CO* and then undergoing another CO_2_ molecular reduction on the Fe site.

## 4 The Role of Nonmetal Moieties in M‐N‐C

**Figure 14:**
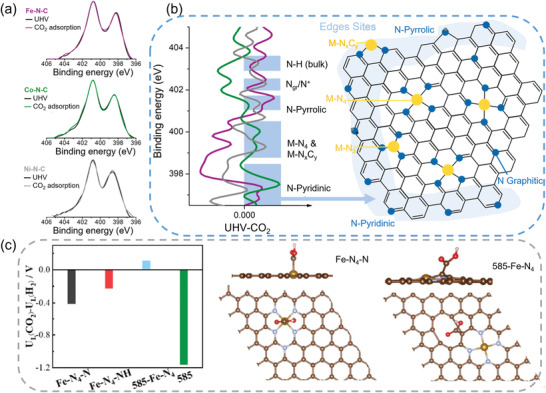
a) N 1s high‐resolution X‐ray photoelectron spectra of the Fe, Co, and Ni‐N‐C under ultrahigh vacuum (UHV) and 13.3 Pa CO_2_ atmosphere. b) Difference between XPS N 1s spectra of the Fe, Co, and Ni‐N‐C electrocatalysts in CO_2_ atmosphere and UHV with the associated structures, and schematic representation of the different preferential adsorption sites for CO_2_ as a function of M (M = Fe, Co, Ni). c) The values of U_L_(CO_2_)‐U_L_(H_2_) for Fe‐N_4_‐N, Fe‐N_4_‐NH, 585‐Fe‐N_4_, and 585‐defect, and view of slab models for *COOH adsorption on Fe‐N_4_‐N and 585‐Fe‐N_4_ site. a,b) Adapted with permission.^[^ ^106^ ^]^ Copyright 2019, American Chemical Society. c) Reproduced with permission.^[^ ^109^ ^]^ Copyright 2020, WILEY‐VCH.

In addition to single metal centers, nonmetal moieties in the carbon plane of M‐N‐C (e.g., N‐doped sites and intrinsic defects) may also serve as active sites for CO_2_RR. Over the past few years, metal‐free N doped carbon catalysts have shown good performance for CO_2_RR, and the pyridinic‐N and/or graphitic‐N defects were identified as the active sites.^[^ ^38^, ^39^, ^40^, ^41^ ^]^ Some studies have also shown that the introduction of metal to prepare M‐N‐C catalysts can further increase the CO_2_RR activity.^[^ ^45^, ^46^, ^47^, ^48^ ^]^ However, the activity of N functionalities in M‐N‐C catalysts cannot be ignored. The role of N moieties on CO_2_RR was studied by Strasser et al. through the preparation of a series of catalysts with the addition of secondary nitrogen precursors to polyaniline‐based FeNC materials.^[^ ^105^ ^]^ They found that both Fe‐N moieties and pyridinic N were likely active sites when comparing specific current density with X‐ray photoelectron spectroscopy (XPS) data. Additionally, the role of pyridinic and pyrrolic N moieties in M‐N‐C with different central metals was further studied by near ambient pressure XPS (**Figure** **14a**).^[^ ^106^ ^]^ It was revealed that these N‐containing sites were not only the active sites for the catalytic reaction but also preferential adsorption sites for CO_2_, which was dependent on the nature of the M‐N_4_ and M‐N*_x_* C*_y_* moieties and the electronic structure of the carbon surface. According to Figure 14b, the preferential adsorption sites for CO_2_ were quite different on M‐N‐C with distinct central metals. For example, CO_2_ was preferentially adsorbed onto pyridinic N, M‐N*_x_* C*_y_* and MN_4_ sites on Co‐N‐C while onto M‐N*_x_* C*_y_* sites on Ni‐N‐C. In contrast, there was no CO_2_ adsorption observed on M‐N*_x_* C*_y_* and MN_4_ sites on Fe‐N‐C, only CO_2_ adsorption on pyridinic N sites.

Furthermore, the role of intrinsic carbon defects in metal‐free N doped carbon materials or M‐N‐C materials for CO_2_RR has also been investigated.^[^ ^107^, ^108^, ^109^ ^]^ Wang and co‐workers prepared an intrinsic defect‐rich porous carbon embedded with atomically dispersed Fe‐N_4_ sites through high‐temperature pyrolysis of carbon‐rich carbon nitride with Fe salt. Compared to NG‐SAFe (without intrinsic defect sites) and DNG (without Fe‐N_4_ site), the material consisting of rich intrinsic defects with the Fe‐N_4_ center (DNG‐SAFe) reached outstanding performance with a maximum CO selectivity up to 90% and CO partial current density of 33 mA cm^−2^. Consistently, the DFT calculation also indicated that the 585‐Fe‐N_4_ (representing DNG‐SAFe) owned the most positive value of U_L_(CO_2_)‐U_L_(H_2_) compared to other structures (Figure 14c). Consequently, the intrinsic carbon defects instead of Fe‐N_4_ moieties in Fe‐N‐C were determined to be the active sites for CO_2_RR.

## 5 The Role of Metal Nanoparticles

During the preparation of M‐N‐C catalysts under pyrolysis, metal nanoparticles (NPs) are inevitably formed. It is well known that exposed metallic species such as Ni and Fe are prone to produce H_2_ in aqueous solution due to their low Gibbs free energy to adsorb hydrogen intermediate.^[^ ^110^, ^111^ ^]^ Moreover, they may also dissolve to form ions under acidic conditions, not only destroying the structure of the M‐N‐C catalysts, but also competing for electrons with CO_2_ reduction. In this situation, there exists a tradeoff between the high content of single metal atom sites and the formation of metal NPs for CO_2_RR.

**Figure 15:**
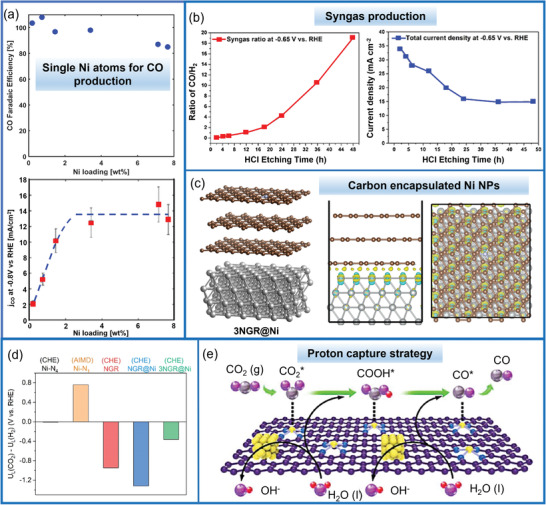
The effect of the ratio of metal single atoms and metal NPs on CO_2_RR performance. a) CO FE and *j*_CO_ at ‐0.8 V versus RHE for a series of Ni‐PACN catalysts with varying Ni loadings (synthesized by changing amount of Ni nitrate added in the synthesis). b) Syngas proportion and total current density as a function of etching time. c) Calculation model and charge density difference graph of 3NGR@Ni (left: side view; right: top view). Yellow and blue represent the charge accumulation and depletion, respectively. d) Difference in limiting potentials for CO_2_ reduction and H_2_ evolution. e) A proposed reaction mechanism for CO production via ECR. a) Reproduced with permission.^[^ ^112^ ^]^ Copyright 2020, WILEY‐VCH. b) Reproduced with permission.^[^ ^114^ ^]^ Copyright 2020, Elsevier B.V. c,d) Reproduced with permission.^[^ ^120^ ^]^ Copyright 2021, WILEY‐VCH. e) Reproduced with permission.^[^ ^121^ ^]^ Copyright 2021, WILEY‐VCH.

Recently, a few works studied the relationship between the metal loading and the overall CO_2_RR performance.^[^ ^112^, ^113^ ^]^ For example, a series of polymer‐derived Ni‐N‐C catalysts with different Ni contents were prepared for CO_2_RR to CO.^[^ ^112^ ^]^ As shown in **Figure** **15a**, it can be observed that CO FE maintains a relatively high value (>80%) with Ni loadings in the range of 0–8 wt%, and drops slightly under high loading situation, which may be due to the appearance of Ni NPs. But the *j*_CO_ rises with Ni content up to ≈2 wt% and then keeps unchanged at 14 mA cm^‐2^ with formation of Ni NPs. These results suggest that the dispersed Ni atoms are responsible for the highly active CO_2_RR in Ni SACs instead of Ni NPs, and the formation of Ni NPs under high Ni loadings did not influence CO_2_RR performance too much as most of the Ni NPs were coated by a thin layer of carbon shells. The adverse influence of Ni NPs on CO_2_RR was also studied by another work, which showed that excess Ni NPs led to enhanced HER.^[^ ^113^ ^]^

Acid washing is commonly adopted to remove unstable metal NPs during SACs synthesis. Recently, self‐supported catalysts containing abundant single Ni atoms before (F‐CPs) and after acid leaching (H‐CPs) were prepared through a solid diffusion approach, and then their CO_2_RR performances were studied.^[^ ^87^ ^]^ H‐CPs exhibited a much higher CO FE over 90% across a broad potential range than the F‐CPs, indicating inactive Ni NPs for CO_2_RR. Furthermore, KSCN poisoning experiment was conducted to show that the exposed isolated Ni sites indeed mainly contributed to the excellent performance for CO_2_ electroreduction to CO. In short, these results provide an insight that when preparing M‐N‐C electrocatalysts, the content of single‐atom sites should be increased as much as possible along with avoiding the formation of metal nanoparticles to enhance the CO_2_RR activity and at the same time suppress HER. Nevertheless, there comes another idea that M‐N‐C containing both the single metal atoms and nanoparticles can be directly applied for syngas synthesis via CO_2_RR in an aqueous solution.^[^ ^114^, ^115^ ^]^ Compared with CO_2_ electroreduction, CO_2_ and water coelectroreduction into syngas is more practical as syngas has been extensively used for industrial production of various hydrocarbons. A series of mesoporous N‐doped carbon nanorods containing both single Ni atoms and Ni NPs were synthesized and used for controlled syngas production from CO_2_ and water.^[^ ^114^ ^]^ The syngas with different ratios of CO to H_2_ could be varied from 1:9 to 19:1 by changing the ratio of single Ni atoms to Ni NPs from 1:4.9 to 1:0 via adjusting the acid leaching time (Figure 15b).

Notably, there are also some works, which proposed that N‐doped carbon encapsulated Ni NPs showed comparable CO_2_RR performance to the single‐atom Ni sites as the inner Ni NPs could tune the electronic structure of the outermost carbon layer.^[^ ^116^, ^117^, ^118^, ^119^ ^]^ However, the existence of single Ni atom sites could not be completely excluded and the excellent electrolytic performance might actually come from the omitted single Ni atom sites. Very recently, we proved that in the Ni‐N‐C system consisting of Ni nanoparticles and single Ni atoms, single Ni atoms should be the real active sites for CO_2_RR instead of N‐doped carbon encapsulated Ni NPs through systematic experiments and theoretical calculations.^[^ ^120^ ^]^ DFT results indicated that the nitrogen‐doped carbon‐encapsulated Ni NPs were selective for the HER rather than for CO_2_ electroreduction, and a synergistic effect between the encapsulated Ni NPs and the surface carbon layer was unlikely exist in the N‐doped carbon‐encapsulated Ni NPs catalysts with more than three carbon layers (Figure 15c,d).

Furthermore, a proton capture strategy by transition‐metal nanoparticles adjacent to atomically dispersed Ni‐N*_x_* sites was proposed to accelerate proton transfer to the latter to improve CO_2_RR, in which a hybrid Ni@NiNCM catalyst containing both Ni NPs and atomically dispersed Ni‐N*_x_* sites on supported carbon was prepared (Figure 15e).^[^ ^121^ ^]^ The states around the Fermi level showed that the Ni@NiN_4_CM possessed a superior electron transport capability to NiN_4_CM, thus leading to enhanced CO_2_RR activity. This proton capture strategy was extendable to other NPs@TM‐NC catalysts.

## 6 Summary and Outlook

Combining electrochemical CO_2_ reduction with a renewable energy source to produce valuable chemicals/fuels offers a promising way to realize carbon recycle as well as alleviate the energy crisis. Recently, M‐N‐C catalysts have shown high efficiency for CO_2_‐to‐CO conversion with high selectivity of up to 100%. In this review, regulation of the active center as well as the local atomic environments in M‐N‐C catalysts to adjust CO_2_RR performance has been discussed in terms of experimental and theoretical findings. A direct view of the performance comparison of previously reported M‐N‐C catalysts with various central metal atoms is provided. Ni‐N‐C tends to be highly selective to produce CO across a wide potential range while Fe‐N‐C possesses a low overpotential for CO formation. Considering the trade‐off between energy input and product yield, Ni‐N‐C and Fe‐N‐C are the most promising candidates for future electrochemical CO_2_RR application at the industrial scale. Furthermore, the catalysts’ performance can also be controlled by tuning the coordination environment of the central metal atoms. But the intrinsic activity of the active sites in M‐N‐C catalysts are still controversial: some reports regarded MN_4_ moieties as the active sites while others suggested a higher activity of unsaturated MN*_x_* moieties; some studies believed that the Ni coordinated with carbon could function as the active site,^[^ ^122^ ^]^ and the intrinsic defect instead of the FeN_4_ sites served as the active site for CO_2_RR was also reported.^[^ ^109^ ^]^ In all, there are still lots of rooms to explore the potential of central metal atoms and coordination atoms to improve the CO_2_RR. Here, some outlooks and challenges are proposed:

(1) In terms of electrocatalysts, although the M‐N‐C catalysts have shown high selectivity up to >90% for CO formation, their activities corresponding to current densities still need to be further improved for large‐scale applications. In this review, some strategies such as the MOF‐assisted approach, in situ thermal transformation approach and solid diffusion method have been included, but more scalable strategies should be invented to synthesize catalysts in facile way and large scales. Besides, electrocatalysts with high surface area and dense single‐atom sites are desired to maximize the amount of active sites accessible to reactant to further improve the activity (current density).
(2) Developing in situ characterization techniques such as Raman spectroscopy, surface‐enhanced infrared absorption spectroscopy, X‐ray absorption and new theoretical calculation approaches are essential to understand the reaction mechanism and speed up the catalyst design. Many studies applied the “Computational Hydrogen Electrode (CHE)” model for DFT calculation. The proposed active sites sometimes, however, are actually not active or selective for CO_2_ reduction according to the calculation results in a strict sense.^[^ ^123^ ^]^ Recently, Liu et al. introduced the surface charge and some layers of water into the simulation and applied ab initio molecular dynamics (AIMD) with the “slow‐growth” method to analyze the kinetic barriers. We believe that in future, more rigorous calculation models like this should be widely used.
(3) Design of flow cells with high mass transfer efficiency is equally significant for further industrial application, for which different architectures have been reviewed and widely applied for CO_2_ electroreduction.^[^ ^124^, ^125^, ^126^ ^]^ Recently, there were some reports on M‐N‐C catalysts for large‐scale CO_2_ electroreduction at industrial current densities adopting the flow cell, which all achieved high CO partial current densities above 100 mA cm^‐2^.^[^ ^69^, ^72^, ^88^, ^127^ ^]^ These results prove that M‐N‐C catalysts are highly promising for efficient CO_2_ electroreduction to CO in industrial scale. In addition to selective CO formation, direct production of syngas (CO+H_2_) that combines CO_2_RR and HER, is also considered as a promising direction for future industrial application. However, the electrodes stability (>1000 h) for CO or syngas production in flow cell system is urgent to be overcome. Furthermore, the energy conversion efficiency of CO_2_RR system should be considered.^[^ ^128^ ^]^ In the future, the optimization of the commercial CO_2_‐recycling system design is one of the major research goals, with voltage, energy utilization efficiency, and lifetime of the electrolyzer being taken into consideration.

## Conflict of Interest

The authors declare no conflict of interest.

## Supporting information

Supporting Information

## References

[1] C. McGlade , P. Ekins , Nature 2015, 517, 187. 25567285 10.1038/nature14016

[2] W. L. Gao , S. Y. Liang , R. J. Wang , Q. Jiang , Y. Zhang , Q. W. Zheng , B. Q. Xie , C. Y. Toe , X. C. Zhu , J. Y. Wang , L. Huang , Y. S. Gao , Z. Wang , C. Jo , Q. Wang , L. D. Wang , Y. F. Liu , B. Louis , J. Scott , A. C. Roger , R. Amal , H. Heh , S. E. Park , Chem. Soc. Rev. 2020, 49, 8584. 33073812 10.1039/d0cs00025f

[3] B. M. Tackett , E. Gomez , J. G. Chen , Nat. Catal. 2019, 2, 381.

[4] X. X. Chang , T. Wang , J. L. Gong , Energy Environ. Sci. 2016, 9, 2177.

[5] S. Xu , E. A. Carter , Chem. Rev. 2019, 119, 6631. 30561988 10.1021/acs.chemrev.8b00481

[6] D. D. Zhu , J. L. Liu , S. Z. Qiao , Adv. Mater. 2016, 28, 3423. 26996295 10.1002/adma.201504766

[7] J. L. Qiao , Y. Y. Liu , F. Hong , J. J. Zhang , Chem. Soc. Rev. 2014, 43, 631. 24186433 10.1039/c3cs60323g

[8] A. Schwarz , R. W. Dodson , J. Phys. Chem. 1989, 93, 409.

[9] N. Han , Y. Wang , H. Yang , J. Deng , J. Wu , Y. Li , Y. Li , Nat. Commun. 2018, 9, 1320. 29615621 10.1038/s41467-018-03712-z PMC5882965

[10] X. Li , W. Bi , M. Chen , Y. Sun , H. Ju , W. Yan , J. Zhu , X. Wu , W. Chu , C. Wu , Y. Xie , J. Am. Chem. Soc. 2017, 139, 14889. 28992701 10.1021/jacs.7b09074

[11] H. Yang , N. Han , J. Deng , J. H. Wu , Y. Wang , Y. P. Hu , P. Ding , Y. F. Li , Y. G. Li , J. Lu , Adv. Energy Mater. 2018, 8, 6.

[12] Y. Hou , Y.‐L. Liang , P.‐C. Shi , Y.‐B. Huang , R. Cao , Appl. Catal., B 2020, 271, 118929.

[13] W. Chen , Z. L. Fan , X. L. Pan , X. H. Bao , J. Am. Chem. Soc. 2008, 130, 9414. 18576652 10.1021/ja8008192

[14] T. Zheng , K. Jiang , H. Wang , Adv. Mater. 2018, 30, 1802066. 10.1002/adma.201802066 30129273

[15] S. M. Lee , H. Lee , J. Kim , S. H. Ahn , S. T. Chang , Appl. Catal., B 2019, 259, 118045.

[16] D. L. T. Nguyen , Y. Kim , Y. J. Hwang , D. H. Won , Carbon Energy 2020, 2, 72.

[17] S. Q. Liu , S. W. Wu , M. R. Gao , M. S. Li , X. Z. Fu , J. L. Luo , ACS Sustainable Chem. Eng. 2019, 7, 14443.

[18] W. Luo , J. Zhang , M. Li , A. Züttel , ACS Catal. 2019, 9, 3783.

[19] A. Wang , J. Li , T. Zhang , Nat. Rev. Chem. 2018, 2, 65.

[20] S. Yang , J. Kim , Y. J. Tak , A. Soon , H. Lee , Angew. Chem., Int. Ed. 2016, 55, 2058. 10.1002/anie.201509241 26710326

[21] C. Z. Zhu , S. F. Fu , Q. R. Shi , D. Du , Y. H. Lin , Angew. Chem., Int. Ed. 2017, 56, 13944. 10.1002/anie.201703864 28544221

[22] Y. Cheng , S. Yang , S. P. Jiang , S. Wang , Small Methods 2019, 3, 1800440.

[23] C. Xu , A. Vasileff , Y. Zheng , S. Z. Qiao , Adv. Mater. Interfaces 2020, 8, 2001904.

[24] X. Su , X. F. Yang , Y. Huang , B. Liu , T. Zhang , Acc. Chem. Res. 2019, 52, 656. 30512920 10.1021/acs.accounts.8b00478

[25] M. Li , H. Wang , W. Luo , P. C. Sherrell , J. Chen , J. Yang , Adv. Mater. 2020, 32, 2001848. 10.1002/adma.202001848 32644259

[26] K. P. Kuhl , T. Hatsukade , E. R. Cave , D. N. Abram , J. Kibsgaard , T. F. Jaramillo , J. Am. Chem. Soc. 2014, 136, 14107. 25259478 10.1021/ja505791r

[27] H. A. Hansen , J. B. Varley , A. A. Peterson , J. K. Norskov , J. Phys. Chem. Lett. 2013, 4, 388. 26281729 10.1021/jz3021155

[28] A. A. Peterson , J. K. Norskov , J. Phys. Chem. Lett. 2012, 3, 251.

[29] C. Rogers , W. S. Perkins , G. Veber , T. E. Williams , R. R. Cloke , F. R. Fischer , J. Am. Chem. Soc. 2017, 139, 4052. 28234002 10.1021/jacs.6b12217

[30] J. Rosen , G. S. Hutchings , Q. Lu , S. Rivera , Y. Zhou , D. G. Vlachos , F. Jiao , ACS Catal. 2015, 5, 4293.

[31] D. F. Gao , H. Zhou , J. Wang , S. Miao , F. Yang , G. X. Wang , J. G. Wang , X. H. Bao , J. Am. Chem. Soc. 2015, 137, 4288. 25746233 10.1021/jacs.5b00046

[32] J. Rosen , G. S. Hutchings , Q. Lu , R. V. Forest , A. Moore , F. Jiao , ACS Catal. 2015, 5, 4586.

[33] J. Q. Zeng , K. Bejtka , W. B. Ju , M. Castellino , A. Chiodoni , A. Sacco , M. A. Farkhondehfal , S. Hernandez , D. Rentsch , C. Battaglia , C. F. Pirri , Appl. Catal., B 2018, 236, 475.

[34] S. Rasul , D. H. Anjum , A. Jedidi , Y. Minenkov , L. Cavallo , K. Takanabe , Angew. Chem., Int. Ed. 2015, 54, 2146. 10.1002/anie.201410233 25537315

[35] C. G. Hu , L. M. Dai , Adv. Mater. 2017, 29, 9. 10.1002/adma.201603486 27982475

[36] Y. W. Ju , S. Yoo , C. Kim , S. Kim , I. Y. Jeon , J. Shin , J. B. Baek , G. Kim , Adv. Sci. 2016, 3, 5. 10.1002/advs.201500205 PMC5049621 27722079

[37] N. R. Sahraie , U. I. Kramm , J. Steinberg , Y. Zhang , A. Thomas , T. Reier , J.‐P. Paraknowitsch , P. Strasser , Nat. Commun. 2015, 6. 10.1038/ncomms9618 PMC4639811 26486465

[38] J. Wu , R. M. Yadav , M. Liu , P. P. Sharma , C. S. Tiwary , L. Ma , X. Zou , X.‐D. Zhou , B. I. Yakobson , J. Lou , P. M. Ajayan , ACS Nano 2015, 9, 5364. 25897553 10.1021/acsnano.5b01079

[39] J. Wu , S. Ma , J. Sun , J. I. Gold , C. Tiwary , B. Kim , L. Zhu , N. Chopra , I. N. Odeh , R. Vajtai , A. Z. Yu , R. Luo , J. Lou , G. Ding , P. J. Kenis , P. M. Ajayan , Nat. Commun. 2016, 7, 13869. 27958290 10.1038/ncomms13869 PMC5159826

[40] S. Liu , H. Yang , X. Huang , L. Liu , W. Cai , J. Gao , X. Li , T. Zhang , Y. Huang , B. Liu , Adv. Funct. Mater. 2018, 28, 1800499.

[41] P. P. Sharma , J. Wu , R. M. Yadav , M. Liu , C. J. Wright , C. S. Tiwary , B. I. Yakobson , J. Lou , P. M. Ajayan , X. D. Zhou , Angew. Chem., Int. Ed. 2015, 54, 13701. 10.1002/anie.201506062 26404732

[42] J. D. Froehlich , C. P. Kubiak , J. Am. Chem. Soc. 2015, 137, 3565. 25714353 10.1021/ja512575v

[43] J. Shen , R. Kortlever , R. Kas , Y. Y. Birdja , O. Diaz‐Morales , Y. Kwon , I. Ledezma‐Yanez , K. J. P. Schouten , G. Mul , M. T. M. Koper , Nat. Commun. 2015,6, 1. 10.1038/ncomms9177 PMC4569799 26324108

[44] S. Lin , C. S. Diercks , Y.‐B. Zhang , N. Kornienko , E. M. Nichols , Y. Zhao , A. R. Paris , D. Kim , P. Yang , O. M. Yaghi , C. J. Chang , Science 2015, 349, 1208. 26292706 10.1126/science.aac8343

[45] Z. Geng , Y. Cao , W. Chen , X. Kong , Y. Liu , T. Yao , Y. Lin , Appl. Catal., B 2019, 240, 234.

[46] J. Gu , C.‐S. Hsu , L. Bai , H. M. Chen , X. Hu , Science 2019, 364, 1091. 31197014 10.1126/science.aaw7515

[47] W. Ju , A. Bagger , G. P. Hao , A. S. Varela , I. Sinev , V. Bon , B. Roldan Cuenya , S. Kaskel , J. Rossmeisl , P. Strasser , Nat. Commun. 2017, 8, 944. 29038491 10.1038/s41467-017-01035-z PMC5643516

[48] A. S. Varela , N. Ranjbar Sahraie , J. Steinberg , W. Ju , H.‐S. Oh , P. Strasser , Angew. Chem., Int. Ed. 2015, 54, 10758. 10.1002/anie.201502099 26227677

[49] S. Ren1 , D. Joulié , D. Salvatore , K. Torbensen , M. Wang , M. Robert , C. P. Berlinguette , Science 2019, 365, 367. 31346062 10.1126/science.aax4608

[50] S. Liu , H. B. Yang , S. F. Hung , J. Ding , W. Cai , L. Liu , J. Gao , X. Li , X. Ren , Z. Kuang , Y. Huang , T. Zhang , B. Liu , Angew. Chem., Int. Ed. 2020, 59, 798. 10.1002/anie.201911995 31657106

[51] Z. Zhang , J. Xiao , X.‐J. Chen , S. Yu , L. Yu , R. Si , Y. Wang , S. Wang , X. Meng , Y. Wang , Z.‐Q. Tian , D. Deng , Angew. Chem., Int. Ed. 2018, 57, 16339. 10.1002/anie.201808593 30312507

[52] F. Pan , W. Deng , C. Justiniano , Y. Li , Appl. Catal., B 2018, 226, 463.

[53] L. Takele Menisa , P. Cheng , C. Long , X. Qiu , Y. Zheng , J. Han , Y. Zhang , Y. Gao , Z. Tang , Nanoscale 2020, 12, 16617. 32756715 10.1039/d0nr03044a

[54] X.‐M. Hu , H. H. Hval , E. T. Bjerglund , K. J. Dalgaard , M. R. Madsen , M.‐M. Pohl , E. Welter , P. Lamagni , K. B. Buhl , M. Bremholm , M. Beller , S. U. Pedersen , T. Skrydstrup , K. Daasbjerg , ACS Catal. 2018, 8, 6255.

[55] L. Jiao , W. Yang , G. Wan , R. Zhang , X. Zheng , H. Zhou , S.‐H. Yu , H.‐L. Jiang , Angew. Chem., Int. Ed. 2020, 59, 20589. 10.1002/anie.202008787 32721058

[56] H. Yang , L. Shang , Q. Zhang , R. Shi , G. I. N. Waterhouse , L. Gu , T. Zhang , Nat. Commun. 2019, 10, 4585. 31594928 10.1038/s41467-019-12510-0 PMC6783464

[57] Z. Chen , K. Mou , S. Yao , L. Liu , ChemSusChem 2018, 11, 2944. 29956488 10.1002/cssc.201800925

[58] K. Zhao , X. Nie , H. Wang , S. Chen , X. Quan , H. Yu , W. Choi , G. Zhang , B. Kim , J. G. Chen , Nat. Commun. 2020, 11, 2455. 32415075 10.1038/s41467-020-16381-8 PMC7229121

[59] A. Guan , Z. Chen , Y. Quan , C. Peng , Z. Wang , T.‐K. Sham , C. Yang , Y. Ji , L. Qian , X. Xu , G. Zheng , ACS Energy Lett. 2020, 5, 1044.

[60] D. Karapinar , N. T. Huan , N. Ranjbar Sahraie , J. Li , D. Wakerley , N. Touati , S. Zanna , D. Taverna , L. H. G. Tizei , A. Zitolo , F. Jaouen , V. Mougel , M. Fontecave , Angew. Chem., Int. Ed. 2019, 131, 15242. 10.1002/anie.201907994 31453650

[61] C. Lu , J. Yang , S. Wei , S. Bi , Y. Xia , M. Chen , Y. Hou , M. Qiu , C. Yuan , Y. Su , F. Zhang , H. Liang , X. Zhuang , Adv. Funct. Mater. 2019, 29, 1806884.

[62] H. B. Yang , S.‐F. Hung , S. Liu , K. Yuan , S. Miao , L. Zhang , X. Huang , H.‐Y. Wang , W. Cai , R. Chen , J. Gao , X. Yang , W. Chen , Y. Huang , H. M. Chen , C. M. Li , T. Zhang , B. Liu , Nat. Energy 2018, 3, 140.

[63] F. Pan , H. Zhang , Z. Liu , D. Cullen , K. Liu , K. More , G. Wu , G. Wang , Y. Li , J. Mater. Chem. A 2019, 7, 26231.

[64] H. Y. Jeong , M. Balamurugan , V. S. K. Choutipalli , J. Jo , H. Baik , V. Subramanian , M. Kim , U. Sim , K. T. Nam , Chem. ‐ Eur. J. 2018, 24, 18444. 30133021 10.1002/chem.201803615

[65] S. Zhao , Y. Cheng , J.‐P. Veder , B. Johannessen , M. Saunders , L. Zhang , C. Liu , M. F. Chisholm , R. De Marco , J. Liu , S.‐Z. Yang , S. P. Jiang , ACS Appl. Energy Mater. 2018, 1, 5286.

[66] Y. Zheng , J. Han , L. Takele , F. Xie , Y. Zhang , J. Sun , B. Han , J. Chen , Y. Gao , Z. Tang , Inorg. Chem. Front. 2019, 6, 1729.

[67] C.‐Z. Yuan , K. Liang , X.‐M. Xia , Z. K. Yang , Y.‐F. Jiang , T. Zhao , C. Lin , T.‐Y. Cheang , S.‐L. Zhong , A.‐W. Xu , Catal. Sci. Technol. 2019, 9, 3669.

[68] Y. J. Sa , H. Jung , D. Shin , H. Y. Jeong , S. Ringe , H. Kim , Y. J. Hwang , S. H. Joo , ACS Catal. 2020, 10, 10920.

[69] Z. Chen , X. Zhang , W. Liu , M. Jiao , K. Mou , X. Zhang , L. Liu , Energy Environ. Sci. 2021, 14, 2349.

[70] S.‐G. Han , D.‐D. Ma , S.‐H. Zhou , K. Zhang , W.‐B. Wei , Y. Du , X.‐T. Wu , Q. Xu , R. Zou , Q.‐L. Zhu , Appl. Catal., B 2021, 283, 119591.

[71] C. Zhao , X. Dai , T. Yao , W. Chen , X. Wang , J. Wang , J. Yang , S. Wei , Y. Wu , Y. Li , J. Am. Chem. Soc. 2017, 139, 8078. 28595012 10.1021/jacs.7b02736

[72] K. Jiang , S. Siahrostami , T. Zheng , Y. Hu , S. Hwang , E. Stavitski , Y. Peng , J. Dynes , M. Gangisetty , D. Su , K. Attenkofer , H. Wang , Energy Environ. Sci. 2018, 11, 893.

[73] C. Yan , H. Li , Y. Ye , H. Wu , F. Cai , R. Si , J. Xiao , S. Miao , S. Xie , F. Yang , Y. Li , G. Wang , X. Bao , Energy Environ. Sci. 2018, 11, 1204.

[74] K. Mou , Z. Chen , X. Zhang , M. Jiao , X. Zhang , X. Ge , W. Zhang , L. Liu , Small 2019, 15, 1903668. 10.1002/smll.201903668 31647616

[75] Q. Fan , P. Hou , C. Choi , T. S. Wu , S. Hong , F. Li , Y. L. Soo , P. Kang , Y. Jung , Z. Sun , Adv. Energy Mater. 2019, 10, 1903068.

[76] J. Yang , Z. Qiu , C. Zhao , W. Wei , W. Chen , Z. Li , Y. Qu , J. Dong , J. Luo , Z. Li , Y. Wu , Angew. Chem., ‐Int. Ed. 2018, 57, 14095. 10.1002/anie.201808049 30203573

[77] Y. Cheng , S. Zhao , H. Li , S. He , J.‐P. Veder , B. Johannessen , J. Xiao , S. Lu , J. Pan , M. F. Chisholm , S.‐Z. Yang , C. Liu , J. G. Chen , S. P. Jiang , Appl. Catal., B 2019, 243, 294.

[78] Y.‐N. Gong , L. Jiao , Y. Qian , C.‐Y. Pan , L. Zheng , X. Cai , B. Liu , S.‐H. Yu , H.‐L. Jiang , Angew. Chem., Int. Ed. 2020, 59, 2705. 10.1002/anie.201914977 31821685

[79] X. Rong , H. J. Wang , X. L. Lu , R. Si , T. B. Lu , Angew. Chem., Int. Ed. 2020, 59, 1961. 10.1002/anie.201912458 31674119

[80] Y. Zhang , L. Jiao , W. Yang , C. Xie , H. L. Jiang , Angew. Chem., Int. Ed. 2021, 60, 7607. 10.1002/anie.202016219 33432715

[81] C. Zhang , S. Yang , J. Wu , M. Liu , S. Yazdi , M. Ren , J. Sha , J. Zhong , K. Nie , A. S. Jalilov , Z. Li , H. Li , B. I. Yakobson , Q. Wu , E. Ringe , H. Xu , P. M. Ajayan , J. M. Tour , Adv. Energy Mater. 2018, 8, 1703487.

[82] J. Feng , H. Gao , L. Zheng , Z. Chen , S. Zeng , C. Jiang , H. Dong , L. Liu , S. Zhang , X. Zhang , Nat. Commun. 2020, 11, 4341. 32859931 10.1038/s41467-020-18143-y PMC7455739

[83] H. Zhang , J. Li , S. Xi , Y. Du , X. Hai , J. Wang , H. Xu , G. Wu , J. Zhang , J. Lu , J. Wang , Angew. Chem., Int. Ed. 2019, 58, 14871. 10.1002/anie.201906079 31368619

[84] Y. Pan , R. Lin , Y. Chen , S. Liu , W. Zhu , X. Cao , W. Chen , K. Wu , W. C. Cheong , Y. Wang , L. Zheng , J. Luo , Y. Lin , Y. Liu , C. Liu , J. Li , Q. Lu , X. Chen , D. Wang , Q. Peng , C. Chen , Y. Li , J. Am. Chem. Soc. 2018, 140, 4218. 29517907 10.1021/jacs.8b00814

[85] H. Chen , X. Guo , X. Kong , Y. Xing , Y. Liu , B. Yu , Q.‐X. Li , Z. Geng , R. Si , J. Zeng , Green Chem. 2020, 22, 7529.

[86] X. Wang , Z. Chen , X. Zhao , T. Yao , W. Chen , R. You , C. Zhao , G. Wu , J. Wang , W. Huang , J. Yang , X. Hong , S. Wei , Y. Wu , Y. Li , Angew. Chem., Int. Ed. 2018, 57, 1944. 10.1002/anie.201712451 29266615

[87] C. Zhao , Y. Wang , Z. Li , W. Chen , Q. Xu , D. He , D. Xi , Q. Zhang , T. Yuan , Y. Qu , J. Yang , F. Zhou , Z. Yang , X. Wang , J. Wang , J. Luo , Y. Li , H. Duan , Y. Wu , Y. Li , Joule 2019, 3, 584.

[88] T. Möller , W. Ju , A. Bagger , X. Wang , F. Luo , T. Ngo Thanh , A. S. Varela , J. Rossmeisl , P. Strasser , Energy Environ. Sci. 2019, 12, 640.

[89] P. Su , K. Iwase , T. Harada , K. Kamiya , S. Nakanishi , Chem. Sci. 2018, 9, 3941. 29780526 10.1039/c8sc00604k PMC5941196

[90] L. Tao , C.‐Y. Lin , S. Dou , S. Feng , D. Chen , D. Liu , J. Huo , Z. Xia , S. Wang , Nano Energy 2017, 41, 417.

[91] W. Zheng , J. Yang , H. Chen , Y. Hou , Q. Wang , M. Gu , F. He , Y. Xia , Z. Xia , Z. Li , B. Yang , L. Lei , C. Yuan , Q. He , M. Qiu , X. Feng , Adv. Funct. Mater. 2019, 30, 1907658.

[92] F. Pan , H. Zhang , K. Liu , D. Cullen , K. More , M. Wang , Z. Feng , G. Wang , G. Wu , Y. Li , ACS Catal. 2018, 8, 3116.

[93] Y. Chen , S. Ji , S. Zhao , W. Chen , J. Dong , W. C. Cheong , R. Shen , X. Wen , L. Zheng , A. I. Rykov , S. Cai , H. Tang , Z. Zhuang , C. Chen , Q. Peng , D. Wang , Y. Li , Nat. Commun. 2018, 9, 5422. 30575726 10.1038/s41467-018-07850-2 PMC6303331

[94] Z. Jiang , W. Sun , H. Shang , W. Chen , T. Sun , H. Li , J. Dong , J. Zhou , Z. Li , Y. Wang , R. Cao , R. Sarangi , Z. Yang , D. Wang , J. Zhang , Y. Li , Energy Environ. Sci. 2019, 12, 3508.

[95] J. Zhang , Y. Zhao , C. Chen , Y. C. Huang , C. L. Dong , C. J. Chen , R. S. Liu , C. Wang , K. Yan , Y. Li , G. Wang , J. Am. Chem. Soc. 2019, 141, 20118. 31804069 10.1021/jacs.9b09352

[96] Y. Han , Y. Wang , R. Xu , W. Chen , L. Zheng , A. Han , Y. Zhu , J. Zhang , H. Zhang , J. Luo , C. Chen , Q. Peng , D. Wang , Y. Li , Energy Environ. Sci. 2018, 11, 2348.

[97] B. Zhang , J. Zhang , J. Shi , D. Tan , L. Liu , F. Zhang , C. Lu , Z. Su , X. Tan , X. Cheng , B. Han , L. Zheng , J. Zhang , Nat. Commun. 2019, 10, 2980. 31278257 10.1038/s41467-019-10854-1 PMC6611886

[98] X. Wang , Y. Wang , X. Sang , W. Zheng , S. Zhang , L. Shuai , B. Yang , Z. Li , J. Chen , L. Lei , N. M. Adli , M. K. H. Leung , M. Qiu , G. Wu , Y. Hou , Angew. Chem., Int. Ed. 2021, 60, 4192. 10.1002/anie.202013427 33197100

[99] J. Wang , W. Liu , G. Luo , Z. Li , C. Zhao , H. Zhang , M. Zhu , Q. Xu , X. Wang , C. Zhao , Y. Qu , Z. Yang , T. Yao , Y. Li , Y. Lin , Y. Wu , Y. Li , Energy Environ. Sci. 2018, 11, 3375.

[100] R. Zhao , Z. Liang , S. Gao , C. Yang , B. Zhu , J. Zhao , C. Qu , R. Zou , Q. Xu , Angew. Chem., Int. Ed. 2019, 58, 1975. 10.1002/anie.201811126 30520258

[101] W. Ren , X. Tan , W. Yang , C. Jia , S. Xu , K. Wang , S. C. Smith , C. Zhao , Angew. Chem., Int. Ed. 2019, 58, 6972. 10.1002/anie.201901575 30920151

[102] M. Xiao , H. Zhang , Y. Chen , J. Zhu , L. Gao , Z. Jin , J. Ge , Z. Jiang , S. Chen , C. Liu , W. Xing , Nano Energy 2018, 46, 396.

[103] X. Yang , T. Tat , A. Libanori , J. Cheng , X. Xuan , N. Liu , X. Yang , J. Zhou , A. Nashalian , J. Chen , Mater. Today 2021, 45, 54.

[104] Q. He , D. Liu , J. H. Lee , Y. Liu , Z. Xie , S. Hwang , S. Kattel , L. Song , J. G. Chen , Angew. Chem., Int. Ed. 2020, 59, 3033. 10.1002/anie.201912719 31826317

[105] N. Leonard , W. Ju , I. Sinev , J. Steinberg , F. Luo , A. S. Varela , B. Roldan Cuenya , P. Strasser , Chem. Sci. 2018, 9, 5064. 29938037 10.1039/c8sc00491a PMC5994794

[106] T. Asset , S. T. Garcia , S. Herrera , N. Andersen , Y. Chen , E. J. Peterson , I. Matanovic , K. Artyushkova , J. Lee , S. D. Minteer , S. Dai , X. Pan , K. Chavan , S. Calabrese Barton , P. Atanassov , ACS Catal. 2019, 9, 7668.

[107] W. Wang , L. Shang , G. Chang , C. Yan , R. Shi , Y. Zhao , G. I. N. Waterhouse , D. Yang , T. Zhang , Adv. Mater. 2019, 31, 1808276. 10.1002/adma.201808276 30919523

[108] Q. Wu , J. Gao , J. Feng , Q. Liu , Y. Zhou , S. Zhang , M. Nie , Y. Liu , J. Zhao , F. Liu , J. Zhong , Z. Kang , J. Mater. Chem. A 2020, 8, 1205.

[109] W. Ni , Z. Liu , Y. Zhang , C. Ma , H. Deng , S. Zhang , S. Wang , Adv. Mater. 2021, 33, 2003238. 10.1002/adma.202003238 33241569

[110] J. Du , L. X. Wang , L. Bai , P. P. Zhang , A. L. Song , G. J. Shao , ACS Sustainable Chem. Eng. 2018, 6, 10335.

[111] R. Subbaraman , D. Tripkovic , K. C. Chang , D. Strmcnik , A. P. Paulikas , P. Hirunsit , M. Chan , J. Greeley , V. Stamenkovic , N. M. Markovic , Nat. Mater. 2012, 11, 550. 22561903 10.1038/nmat3313

[112] D. M. Koshy , S. Chen , D. U. Lee , M. B. Stevens , A. M. Abdellah , S. M. Dull , G. Chen , D. Nordlund , A. Gallo , C. Hahn , D. C. Higgins , Z. Bao , T. F. Jaramillo , Angew. Chem., Int. Ed. 2020, 59, 4043. 10.1002/anie.201912857 31919948

[113] C. F. Wen , F. Mao , Y. Liu , X. Y. Zhang , H. Q. Fu , L. R. Zheng , P. F. Liu , H. G. Yang , ACS Catal. 2019, 10, 1086.

[114] W. Zhu , J. Fu , J. Liu , Y. Chen , X. Li , K. Huang , Y. Cai , Y. He , Y. Zhou , D. Su , J.‐J. Zhu , Y. Lin , Appl. Catal., B 2020, 264, 118502.

[115] S. Shen , C. Han , B. Wang , Y. Du , Y. Wang , Appl. Catal., B 2020, 279, 119380.

[116] M. Jia , C. Choi , T.‐S. Wu , C. Ma , P. Kang , H. Tao , Q. Fan , S. Hong , S. Liu , Y.‐L. Soo , Y. Jung , J. Qiu , Z. Sun , Chem. Sci. 2018, 9, 8775. 30746113 10.1039/c8sc03732a PMC6335639

[117] C.‐Z. Yuan , H.‐B. Li , Y.‐F. Jiang , K. Liang , S.‐J. Zhao , X.‐X. Fang , L.‐B. Ma , T. Zhao , C. Lin , A.‐W. Xu , J. Mater. Chem. A 2019, 7, 6894.

[118] W. Zheng , C. Guo , J. Yang , F. He , B. Yang , Z. Li , L. Lei , J. Xiao , G. Wu , Y. Hou , Carbon 2019, 150, 52.

[119] T. Wang , J. Yang , J. Chen , Q. He , Z. Li , L. Lei , J. Lu , M. K. H. Leung , B. Yang , Y. Hou , Chin. Chem. Lett. 2020, 31, 1438.

[120] S. Liang , Q. Jiang , Q. Wang , Y. Liu , Adv. Energy Mater. 2021, 11, 2101477.

[121] X. Wang , X. Sang , C.‐L. Dong , S. Yao , L. Shuai , J. Lu , B. Yang , Z. Li , L. Lei , M. Qiu , L. Dai , Y. Hou , Angew. Chem., Int. Ed. 2021, 60, 2. 10.1002/anie.202100011 33599063

[122] K. Jiang , S. Siahrostami , A. J. Akey , Y. Li , Z. Lu , J. Lattimer , Y. Hu , C. Stokes , M. Gangishetty , G. Chen , Y. Zhou , W. Hill , W.‐B. Cai , D. Bell , K. Chan , J. K. Nørskov , Y. Cui , H. Wang , Chem 2017, 3, 950.

[123] X. Zhao , Y. Liu , J. Am. Chem. Soc. 2020, 142, 5773. 32122132 10.1021/jacs.9b13872

[124] S. Liang , N. Altaf , L. Huang , Y. Gao , Q. Wang , J. CO2 Util. 2020, 35, 90.

[125] D. M. Weekes , D. A. Salvatore , A. Reyes , A. Huang , C. P. Berlinguette , Acc. Chem. Res. 2018, 51, 910. 29569896 10.1021/acs.accounts.8b00010

[126] B. Endrődi , G. Bencsik , F. Darvas , R. Jones , K. Rajeshwar , C. Janáky , Prog. Energy Combust. Sci. 2017, 62, 133.

[127] T. Zheng , K. Jiang , N. Ta , Y. Hu , J. Zeng , J. Liu , H. Wang , Joule 2019, 3, 265.

[128] S. Verma , S. Lu , P. J. A. Kenis , Nat. Energy 2019, 4, 466.

