# Supplementary material for: Electrochemical Reduction of CO2 to CO over Transition Metal/N‐Doped Carbon Catalysts: The Active Sites and Reaction Mechanism
Source: Adv Sci (Weinh). 2021 Oct 31;8(24):2102886. doi: 10.1002/advs.202102886 (PMC8693035; doi:10.1002/advs.202102886)
Supplement: Supplementary file 1 — Supporting Information [file ADVS-8-2102886-s001.pdf]

© 2021 Wiley-VCH GmbH

ADVANCED SCIENCE
Open Access

Supporting Information

for *Adv. Sci.,* DOI: 10.1002/advs.202102886

Electrochemical Reduction of CO_2 to CO over Transition Metal/N-doped Carbon Catalysts: The Active Sites and Reaction Mechanism

Shuyu Liang, Liang Huang, Yanshan Gao, Qiang Wang,, and Bin Liu,**

Supporting Information

Electrochemical Reduction of CO_2 to CO over Transition Metal/N-doped Carbon Catalysts: The Active Sites and Reaction Mechanism

Shuyu Liang, Liang Huang, Yanshan Gao, Qiang Wang,, and Bin Liu,**

S. Liang, Dr. L. Huang, Dr. Y. Gao, Prof. Dr. Q. Wang

College of Environmental Science and Engineering

Beijing Forestry University

35 Qinghua East Road, Haidian District, Beijing 100083, P. R. China

E-mail: qiangwang@bjfu.edu.cn

Prof. Dr. B. Liu

School of Chemical and Biomedical Engineering

Nanyang Technological University

62 Nanyang Drive, Singapore 637459, Singapore

E-mail: liubin@ntu.edu.sg

Table S1. Comparison of CO_2RR performance for different Ni-N-C catalysts examined in H-type cell.

**M-N-C catalyst**	**Active site(s)**	**Metal content (wt. %)**	**Onset potential (vs. RHE)**	**CO FE (%)**	**jCO (mA cm^{-2})**	**TOF (h^{-1})**	**Stability**
Ni-N-Gr[1]	Ni-N_x	2.2	−0.5 V	95 @−0.7 V (*vs*. RHE)	0.2 @−0.65 V (*vs*. RHE)	2700 @−0.7 V (*vs*. RHE)	5 h @−0.65 V (*vs*. RHE)
Ni-N-C [2]	/	/	−0.52 V	84.2 @−0.75 V (*vs*. RHE)	9.3 @−0.75 V (*vs*. RHE)	/	/
Ni-N-C [3]	/	2.83	−0.5 V	96 @−0.76 V (*vs*. RHE)	8.2 @−0.76 V (*vs*. RHE)	1060 @−0.76 V (*vs*. RHE)	10 @−0.75 V (*vs*. RHE)
Ni-N-C [4]	Pyridine-type Ni-N_4	/	−0.4 V	97 @−0.8 V (*vs*. RHE)	17.6 @−0.8 V (*vs*. RHE)	20664 @−0.8 V (*vs*. RHE)	24 h @−0.8 V (*vs*. RHE)
Ni-N-C [5]	Pyridine-type Ni-N_4	0.24	/	93.3 @−0.67 V (*vs*. RHE)	3.64 @−0.67 V (*vs*. RHE)	/	12 h @−0.67 V (*vs*. RHE)
Ni_1-N-C[6]	Pyridine-type	1.75	−0.4 V	96.8 @−0.8 V	27 @−0.8 V	11315 @−0.8 V	10 h @−0.8 V

	$Ni-N_4$			(*vs.* RHE)	(*vs.* RHE)	(*vs.* RHE)	(*vs.* RHE)
Ni-SA[7]	Pyridine-type $Ni-N_4$	2.5	/	98.9 @−1.2 V (*vs.* RHE)	1 @−0.8 V (*vs.* RHE)	/	20 h @−0.8 V (*vs.* RHE)
$Ni-N_5$/HNPCSs[8]	NiN_5	3.32	/	50 @−0.79 V (*vs.* RHE)	/	/	/
$Ni-N_4$-C[9]	Pyridine-type $Ni-N_4$	1.41	−0.4 V	99 @−0.81 V (*vs.* RHE)	28.3 @−0.81 V (*vs.* RHE)	/	30 h @−0.81 V (*vs.* RHE)
Ni-N-RGO[10]	Pyridine-type $Ni-N_4$	1.93	−0.4 V	97 @−0.8 V (*vs.* RHE)	43.9 @−1.1 V (*vs.* RHE)	/	/
NiSA-N-CNT[11]	Pyridine-type $Ni-N_4$	20.3	−0.28 V	91.3 @−0.7 V (*vs.* RHE)	23.5 @−0.7 V (*vs.* RHE)	42120 @−0.55 V (*vs.* RHE)	12 h @−0.7 V (*vs.* RHE)
A-Ni-NG[12]	Pyridine-type $Ni-N_4$	4.6	−0.35 V	97 @−0.72 V (*vs.* RHE)	30.6 @−0.72 V (*vs.* RHE)	8000 @−0.72 V (*vs.* RHE)	/
A-Ni-NSG[12]	Pyridine-type $Ni-N_4$	2.5	−0.25 V	97 @−0.72 V (*vs.*	22 @−0.72 V (*vs.* RHE)	14800 @−0.72 V (*vs.*	100 h @−0.72 V (*vs.*

				RHE)		RHE)	RHE)
Ni/N-Pc[13]	Pyridine-type Ni-N_4	1.69	−0.47 V	84.5 @−0.8 V (*vs.* RHE)	20.9 @−0.9 V (*vs.* RHE)	2029 @−0.9 V (*vs.* RHE)	20 h @−0.9 V (*vs.* RHE)
NiPor-CTF[13]	Pyrrole-type Ni-N_4	2.4	−0.44 V	97 @−0.9 V (*vs.* RHE)	51.3 @−0.9 V (*vs.* RHE)	1690 @−0.9 V (*vs.* RHE)	20 h @−0.9 V (*vs.* RHE)
Ni-N-C [14]	Edge-located Ni-N_{2+2}	0.16 at%	−0.29 V	97 @−0.75 V (*vs.* RHE)	7.51 @−0.75 V (*vs.* RHE)	/	10 h @−0.75 V (*vs.* RHE)
Ni-N-C [15]	Pyridine-type Ni-N_4	1.63	−0.57 V	90 @−0.8 V (*vs.* RHE)	11.6 @−0.8 V (*vs.* RHE)	3079 @−0.8 V (*vs.* RHE)	6 h @−0.8 V (*vs.* RHE)
Ni-N-C [16]	Pyridine-type Ni-N_4	1.25	−0.4 V	91.2 @−0.9 V (*vs.* RHE)	~11.6 @−0.9 V (*vs.* RHE)	/	22 h @−0.9 V (*vs.* RHE)
NiPc/CNT[17]	NiN_4	0.21	/	97.6 @−0.73 V (*vs.* RHE)	3.27 @−0.73 V (*vs.* RHE)	2960 @−0.9 V (*vs.* RHE)	/
Ni-CNT-CC[18]	NiN_4	0.27	−0.36 V	98 @−0.65 V (*vs.* RHE)	17.5 @−0.65 V (*vs.* RHE)	100179 @−0.71 V (*vs.* RHE)	100 h @−0.65 V (*vs.* RHE)

Ni-N_4[19]	Pyridine-type NiN_4	/	−0.5 V	85 @−0.7 V (*vs.* RHE)	7.1 @−0.7 V (*vs.* RHE)	34600 @−0.9 V (*vs.* RHE)	/
Ni SAs/N-C[20]	NiN_3C	1.53	−0.57 V	71.9 @−0.9 V (*vs.* RHE)	7.37 @−1 V (*vs.* RHE)	5273 @−1 V (*vs.* RHE)	60 h @−1 V (*vs.* RHE)
Ni_{SA}-N_2-C[21]	NiN_2C_2	0.9	−0.5 V	98.1 @−0.8 V (*vs.* RHE)	10 @−0.8 V (*vs.* RHE)	1622 @−0.8 V (*vs.* RHE)	10 h @−0.8 V (*vs.* RHE)
Ni_{SA}-N_3-C[21]	NiN_3C	0.9	−0.52 V	90.7 @−0.8 V (*vs.* RHE)	/	1120 @−0.8 V (*vs.* RHE)	/
C-Zn_1Ni_4 ZIF-8[22]	NiN_3 NiN_3V NiN_2V_2	5.44	−0.43 V	98 @−0.83 V (*vs.* RHE)	71.5±2.9 @−1.03 V (*vs.* RHE)	10087 @−1.03 V (*vs.* RHE)	12 h @−0.63 V (*vs.* RHE)
NiSA-NGA[23]	NiN_3V NiN_2V_2	2.6	−0.4 V	90.2 @−0.8 V (*vs.* RHE)	~6 @−0.8 V (*vs.* RHE)	/	6 h @−0.8 V (*vs.* RHE)
Ni-N_3-C[24]	NiN_3V	0.85	−0.42 V	95.6 @−0.65 V (*vs.* RHE)	6.64 @−0.65 V (*vs.* RHE)	1425 @−0.65 V (*vs.* RHE)	10 h @−0.65 V (*vs.* RHE)
H-NiPc	NiN_3V	0.17	/	97	9	13860	16 h

/CNT[17]				@−0.77 V (*vs.* RHE)	@−0.77 V (*vs.* RHE)	@−0.94 V (*vs.* RHE)	@−0.79 V (*vs.* RHE)
Ni-N_3-V[19]	NiN_3V	/	−0.32 V	94 @−0.8 V (*vs.* RHE)	48 @−0.8 V (*vs.* RHE)	135000 @−0.9 V (*vs.* RHE)	14 h @−0.7 V (*vs.* RHE)
NC-CNTs (Ni)[25]	NiN_3	1.01	−0.4 V	90 @−0.8 V (*vs.* RHE)	9.2 @−1 V (*vs.* RHE)	11650 @−0.89 V (*vs.* RHE)	10 h @−0.8 V (*vs.* RHE)
SE-Ni SAs@PNC[26]	NiN_3 NiN_4	/	/	95.7 @−0.8 V (*vs.* RHE)	18.3 @−1 V (*vs.* RHE)	47805 @−1 V (*vs.* RHE)	60 h @−1 V (*vs.* RHE)
Ni-N-MEGO[27]	Unsaturated Ni-N (edge)	6.9	−0.18 V	92.1 @−0.7 V (*vs.* RHE)	26.8 @−0.7 V (*vs.* RHE)	864 @−0.7 V (*vs.* RHE)	20 h @−0.55 V (*vs.* RHE)
Ni-NG[28]	Ni-N@SV	2.07	−0.31 V	95 @−0.73 V (*vs.* RHE)	11 @−0.73 V (*vs.* RHE)	24480 @−0.68 V (*vs.* RHE)	20 h @−0.75 V (*vs.* RHE)
Ni–N_4/C–NH_2[29]	NiN_4	5.32	−0.22 V	96.2 @−0.7 V (*vs.* RHE)	63.6 @−1.0 V (*vs.* RHE)	/	10 h @−0.8 V (*vs.* RHE)
Ni-N_4-O/C[30]	NiN_4O	0.37	−0.41 V	~100 @−0.9 V	23 @−0.9 V	11187 @−0.9 V	20 h @−0.9 V

				(*vs.* RHE)	(*vs.* RHE)	(*vs.* RHE)	(*vs.* RHE)
Ni-SAs @FNC[31]	Pyridine-type NiN_4	5.92	/	96.3 @−0.77 V (*vs.* RHE)	25 @−0.77 V (*vs.* RHE)	/	10 h @−0.77 V (*vs.* RHE)

Table S2. Comparison of CO_2RR performance for different Fe-N-C catalysts examined in H-type cell.

Fe-N-C [2]	/	/	−0.4 V	65 @−0.52 V (*vs.* RHE)	2.15 @−0.52 V (*vs.* RHE)	/	/
Fe-N-C [3]	/	2.14	−0.3 V	87 @−0.49 V (*vs.* RHE)	1.3 @−0.49 V (*vs.* RHE)	410 @−0.58 V (*vs.* RHE)	/
Fe-N-C [4]	Pyridine-type Fe-N_4	/	−0.3 V	86.8 @−0.5 V (*vs.* RHE)	2.7 @−0.5 V (*vs.* RHE)	20664 @−0.5 V (*vs.* RHE)	24 h @−0.5 V (*vs.* RHE)
Fe-N-C [5]	Pyridine-type Fe-N_4	0.4	/	85 @−0.47 V (*vs.* RHE)	1.15 @−0.47 V (*vs.* RHE)	/	12 h @−0.47 V (*vs.* RHE)
Fe_1-N-C[6]	Pyridine-type Fe-N_4	1.78	−0.32 V	86.5 @−0.5 V (*vs.*	2.85 @−0.5 V (*vs.* RHE)	1100 @−0.5 V (*vs.*	/

				RHE)		RHE)	
Fe-SA[7]	Pyridine-type $Fe-N_4$	~2.5	/	77 @−1.2 V (*vs.* RHE)	/	/	/
$Fe-N_4$/CF-1000[32]	$Fe-N_4$	0.2	/	94.9 @−0.5 V (*vs.* RHE)	2.85 @−0.5 V (*vs.* RHE)	700 @−0.5 V (*vs.* RHE)	60 h @−0.5 V (*vs.* RHE)
$Fe-N_4$/CF-900[32]	$Fe-N_4$	0.86	/	84.7 @−0.5 V (*vs.* RHE)	4.27 @−0.5 V (*vs.* RHE)	250 @−0.5 V (*vs.* RHE)	/
$Fe-N_5$/HNPCSs[8]	FeN_5	3.03	/	82 @−0.66 V (*vs.* RHE)	/	/	/
Fe^{2+}-N-C[33]	FeN_xC_y	2.6	/	75.5 @−0.37 V (*vs.* RHE)	0.3 @−0.37 V (*vs.* RHE)	20 @−0.37 V (*vs.* RHE)	/
Fe^{3+}-N-C[33]	FeN_xC_y	2.8	−0.19 V	95 @−0.47 V (*vs.* RHE)	20 @−0.47 V (*vs.* RHE)	1400 @−0.47 V (*vs.* RHE)	12 h @−0.37 V (*vs.* RHE)
Fe-N-C[34]	Edge-located $Fe-N_{2+2}$	0.1 at%	−0.29 V	93 @−0.58 V (*vs.* RHE)	2.6 @−0.58 V (*vs.* RHE)	/	20 h @−0.58 V (*vs.* RHE)

Fe/NG-750[35]	FeN_4	1.25	−0.3 V	80 @−0.57 V (*vs.* RHE)	2 @−0.6 V (*vs.* RHE)	/	10 h @−0.6 V (*vs.* RHE)
Fe-N_5[36]	FeN_5	0.57	−0.16 V	99 @−0.35 V (*vs.* RHE)	20.8 @−1.05 V (*vs.* RHE)	5006 @−1.05 V (*vs.* RHE)	24 h @−0.4 V (*vs.* RHE)
Fe-N_6[36]	FeN_6	0.58	−0.2 V	96 @−0.35 V (*vs.* RHE)	5.5 @−1.05 V (*vs.* RHE)	1324 @−1.05 V (*vs.* RHE)	/
Fe-N_5[37]	FeN_5	0.8 at%	−0.26 V	97 @−0.46 V (*vs.* RHE)	1.81 @−0.46 V (*vs.* RHE)	/	24 h @−0.46 V (*vs.* RHE)
Fe-N_4[37]	FeN_4	/	/	66 @−0.56 V (*vs.* RHE)	0.3 @−0.56 V (*vs.* RHE)	/	/

Table S3. Comparison of CO_2RR performance for different Co-N-C catalysts examined in H-type cell.

Co-N-C	/	/	−0.4 V	17.1 @−0.51 V (*vs.* RHE)	1.15 @−0.51 V (*vs.* RHE)	/	/
Co-N-C	/	2.52	−0.4 V	46	2.7	230	/

[3]				@−0.67 V (*vs*. RHE)	@−0.67 V (*vs*. RHE)	@−0.58 V (*vs*. RHE)	
Co-N-C [4]	Pyridine-type Co-N_4	/	−0.4 V	48.7 @−0.5 V (*vs*. RHE)	0.66 @−0.5 V (*vs*. RHE)	/	/
Co-N-C [5]	Pyridine-type Co-N_4	0.35	/	16.7 @−0.77 V (*vs*. RHE)	2.07 @−0.77 V (*vs*. RHE)	/	/
Co_1-N-C[6]	Pyridine-type Co-N_4	1.57	−0.4 V	17.8 @−0.65 V (*vs*. RHE)	1.1 @−0.65 V (*vs*. RHE)	900 @−0.65 V (*vs*. RHE)	/
Co-SA[7]	Pyridine-type Co-N_4	~2.5	/	90 @−1.2 V (*vs*. RHE)	/	/	/
Co_1-N_4[38]	Pyridine-type Co-N_4	0.6	−0.3 V	82 @−0.8 V (*vs*. RHE)	15.8 @−0.8 V (*vs*. RHE)	1455 @−1.0 V (*vs*. RHE)	10 h @−0.8 V (*vs*. RHE)
Co_1-N_{4-x}[38]	Co-N_x	0.63	−0.32 V	47 @−0.8 V (*vs*. RHE)	8.8 @−0.8 V (*vs*. RHE)	763 @−1.0 V (*vs*. RHE)	10 h @−0.8 V (*vs*. RHE)
Co-N_2[39]	CoN_2C_2	0.25	−0.22 V	94 @−0.63	17 @−0.63 V	18200 @−0.63	60 h @−0.63

				V (*vs.* RHE)	(*vs.* RHE)	V (*vs.* RHE)	V (*vs.* RHE)
Co-N_3[39]	CoN_3C	/	−0.45 V	63 @−0.53 V (*vs.* RHE)	1.56 @−0.53 V (*vs.* RHE)	1250 @−0.63 V (*vs.* RHE)	/
Co-N_4[39]	Pyridine-type CoN_4	/	/	4.2 @−0.83 V (*vs.* RHE)	/	84 @−0.83 V (*vs.* RHE)	/
Co-N_5/HNPCSs[8]	CoN_5	3.54	/	99.4 @−0.79 V (*vs.* RHE)	4.5 @−0.73 V (*vs.* RHE)	480.2 @−0.73 V (*vs.* RHE)	10 h @−0.73 V (*vs.* RHE)
Co-N-C [34]	Edge-located Co-N_{2+2}	0.1 at%	−0.38 V	45 @−0.59 V (*vs.* RHE)	0.8 @−0.59 V (*vs.* RHE)	/	/

Table S4. Comparison of CO_2RR performance for different Mn-N-C catalysts examined in H-type cell.

Mn-N-C[3]	/	2.52	−0.41 V	70 @−0.6 V (*vs.* RHE)	0.4 @−0.6 V (*vs.* RHE)	230 @−0.58 V (*vs.* RHE)	/
Mn-SA[7]	Pyridine-type Mn-N_4	~2.5	/	61 @−1.2 V (*vs.*	/	/	/

				RHE)			
Mn–C_3N_4/CNT[40]	Mn-N_3	0.17	−0.3 V	98.8 @−0.55 V (*vs.* RHE)	14 @−0.55 V (*vs.* RHE)	/	20 h @−0.55 V (*vs.* RHE)
(Cl, N)-Mn/G[41]	MnN_4Cl	0.049	/	97 @−0.6 V (*vs.* RHE)	10 @−0.6 V (*vs.* RHE)	38347 @−0.6 V (*vs.* RHE)	12 h @−0.55 V (*vs.* RHE)

Table S5. Comparison of CO_2RR performance for different Cu-N-C catalysts examined in H-type cell.

Cu-N-C [2]	/	/	−0.5 V	30 @−0.73 V (*vs.* RHE)	2.39 @−0.73 V (*vs.* RHE)	/	/
Cu-N-C [4]	Pyridine-type Cu-N_4	/	/	28.4 @−0.8 V (*vs.* RHE)	2 @−0.8 V (*vs.* RHE)	/	/
Cu_1-N-C[6]	Pyridine-type Cu-N_4	1.43	−0.46 V	14 @−0.6 V (*vs.* RHE)	0.35 @−0.85 V (*vs.* RHE)	/	/
Cu-SA[7]	Pyridine-type Cu-N_4	~2.5	/	12 @−1.2 V (*vs.* RHE)	/	/	/

Cu-N_5/ HNPCSs[8]	CuN_5	3.75	/	2 @−0.73 V (*vs.* RHE)	/	/	/
CuSAs/ TCNFs [42]	Pyridine-type Cu-N_4	1.3	−0.41 V	CO: 56 (methanol: 44%) @−0.9 V (*vs.* RHE)	50.4 @−0.9 V (*vs.* RHE)	/	50 h @−0.9 V (*vs.* RHE)
Cu SAs/N C [43]	Cu-N_4	0.32	−0.23 V	92 @−0.7 V (*vs.* RHE)	8.9 @−1.0 V (*vs.* RHE)	/	30 h @−0.7 V (*vs.* RHE)
Cu-N_2/ GN[44]	CuN_2V_2	1.45	−0.33 V	81 @−0.5 V (*vs.* RHE)	0.73 @−0.5 V (*vs.* RHE)	/	10 h @−0.5 V (*vs.* RHE)
Cu-N_4/ GN-800[44]	Pyridine-type Cu-N_4	1.14	−0.4 V	62 @−0.5 V (*vs.* RHE)	/	/	/

Table S6. Comparison of CO_2RR performance for different Zn-N-C catalysts examined in H-type cell.

Zn-SA[7]	Pyridine-type Zn-N_4	~2.5	/	21 @−1.2 V (*vs.* RHE)	/	/	/

Zn-N-G [45]	Pyridine-type $Zn\text{-}N_4$	0.31 at%	−0.4 V	91 @−0.5 V (*vs.* RHE)	11.2 @−0.8 V (*vs.* RHE)	/	15 h @−0.5 V (*vs.* RHE)
Zn SAs/N-C[46]	Pyridine-type $Zn\text{-}N_4$	0.13	−0.16 V	94.7 @−0.44 V (*vs.* RHE)	10.45 @−0.44 V (*vs.* RHE)	8190 @−0.64 V (*vs.* RHE)	30 h @−0.44 V (*vs.* RHE)
SA-Zn-NHPC[47]	Pyridine-type $Zn\text{-}N_4$	0.34	−0.16 V	96 @−0.44 V (*vs.* RHE)	4.5 @−0.44 V (vs. RHE)	10113 @−0.74 V (vs. RHE)	20 h @−0.44 V (*vs.* RHE)
ZnN_x/C [48]	Pyridine-type $Zn\text{-}N_4$	0.1	−0.134 V	95 @−0.43 V (*vs.* RHE)	4.56 @−0.43 V (*vs.* RHE)	9969 @−0.43 V (*vs.* RHE)	75 h @−0.43 V (*vs.* RHE)

Reference

[1] P. Su, K. Iwase, S. Nakanishi, K. Hashimoto, K. Kamiya, *Small* **2016**, *12*, 6083.

[2] W. Ju, A. Bagger, G. P. Hao, A. S. Varela, I. Sinev, V. Bon, B. Roldan Cuenya, S. Kaskel, J. Rossmeisl, P. Strasser, *Nat. Commun.* **2017**, *8*, 944.

[3] F. Pan, W. Deng, C. Justiniano, Y. Li, *Appl. Catal. B* **2018**, *226*, 463.

[4] L. Takele Menisa, P. Cheng, C. Long, X. Qiu, Y. Zheng, J. Han, Y. Zhang, Y. Gao, Z. Tang, *Nanoscale* **2020**, *12*, 16617.

[5] X.-M. Hu, H. H. Hval, E. T. Bjerglund, K. J. Dalgaard, M. R. Madsen, M.-M. Pohl, E. Welter, P. Lamagni, K. B. Buhl, M. Bremholm, M. Beller, S. U. Pedersen, T. Skrydstrup, K. Daasbjerg, *ACS Catal.* **2018**, *8*, 6255.

[6] Long Jiao, Weijie Yang, Gang Wan, Rui Zhang, Xusheng Zheng, Hua Zhou, Shu-Hong Yu, H.-L. Jiang, *Angew. Chem. Int. Ed.* **2020**, *59*, 20589.

[7] H. Yang, L. Shang, Q. Zhang, R. Shi, G. I. N. Waterhouse, L. Gu, T. Zhang, *Nat. Commun.* **2019**, *10*, 4585.

[8] Y. Pan, R. Lin, Y. Chen, S. Liu, W. Zhu, X. Cao, W. Chen, K. Wu, W. C. Cheong, Y. Wang, L. Zheng, J. Luo, Y. Lin, Y. Liu, C. Liu, J. Li, Q. Lu, X. Chen, D. Wang, Q. Peng, C. Chen, Y. Li, *J. Am. Chem. Soc.* **2018**, *140*, 4218.

[9] X. Li, W. Bi, M. Chen, Y. Sun, H. Ju, W. Yan, J. Zhu, X. Wu, W. Chu, C. Wu, Y. Xie, *J. Am. Chem. Soc.* **2017**, *139*, 14889.
[10] H. Y. Jeong, M. Balamurugan, V. S. K. Choutipalli, J. Jo, H. Baik, V. Subramanian, M. Kim, U. Sim, K. T. Nam, *Chem-Eur. J* **2018**, *24*, 18444.
[11] S. Zhao, Y. Cheng, J.-P. Veder, B. Johannessen, M. Saunders, L. Zhang, C. Liu, M. F. Chisholm, R. De Marco, J. Liu, S.-Z. Yang, S. P. Jiang, *ACS Appl. Energ. Mater.* **2018**, *1*, 5286.
[12] H. B. Yang, S.-F. Hung, S. Liu, K. Yuan, S. Miao, L. Zhang, X. Huang, H.-Y. Wang, W. Cai, R. Chen, J. Gao, X. Yang, W. Chen, Y. Huang, H. M. Chen, C. M. Li, T. Zhang, B. Liu, *Nat. Energy* **2018**, *3*, 140.
[13] C. Lu, J. Yang, S. Wei, S. Bi, Y. Xia, M. Chen, Y. Hou, M. Qiu, C. Yuan, Y. Su, F. Zhang, H. Liang, X. Zhuang, *Adv. Funct. Mater.* **2019**, *29*, 1806884.
[14] F. Pan, H. Zhang, Z. Liu, D. Cullen, K. Liu, K. More, G. Wu, G. Wang, Y. Li, *J. Mater. Chem. A* **2019**, *7*, 26231.
[15] Y. Zheng, J. Han, L. Takele, F. Xie, Y. Zhang, J. Sun, B. Han, J. Chen, Y. Gao, Z. Tang, *Inorg. Chem. Front.* **2019**, *6*, 1729.
[16] C.-Z. Yuan, K. Liang, X.-M. Xia, Z. K. Yang, Y.-F. Jiang, T. Zhao, C. Lin, T.-Y. Cheang, S.-L. Zhong, A.-W. Xu, *Catal. Sci. Technol.* **2019**, *9*, 3669.
[17] Y. J. Sa, H. Jung, D. Shin, H. Y. Jeong, S. Ringe, H. Kim, Y. J. Hwang, S. H. Joo, *ACS Catal.* **2020**, *10*, 10920.
[18] S. Liu, H. B. Yang, S. F. Hung, J. Ding, W. Cai, L. Liu, J. Gao, X. Li, X. Ren, Z. Kuang, Y. Huang, T. Zhang, B. Liu, *Angew. Chem. Int. Ed.* **2020**, *59*, 798.
[19] X. Rong, H. J. Wang, X. L. Lu, R. Si, T. B. Lu, *Angew. Chem. Int. Ed.* **2020**, *59*, 1961.
[20] C. Zhao, X. Dai, T. Yao, W. Chen, X. Wang, J. Wang, J. Yang, S. Wei, Y. Wu, Y. Li, *J. Am. Chem. Soc.* **2017**, *139*, 8078.
[21] Y.-N. Gong, L. Jiao, Y. Qian, C.-Y. Pan, L. Zheng, Xuechao Cai, B. Liu, S.-H. Yu, H.-L. Jiang, *Angew. Chem. Int. Ed.* **2020**, *59*, 2705
[22] C. Yan, H. Li, Y. Ye, H. Wu, F. Cai, R. Si, J. Xiao, S. Miao, S. Xie, F. Yang, Y. Li, G. Wang, X. Bao, *Energy. Environ. Sci.* **2018**, *11*, 1204.
[23] K. Mou, Z. Chen, X. Zhang, M. Jiao, X. Zhang, X. Ge, W. Zhang, L. Liu, *Small* **2019**, *15*, 1903668.
[24] Y. Zhang, L. Jiao, W. Yang, C. Xie, H. L. Jiang, *Angew. Chem. Int. Ed.* **2021**, *60*, 7607.
[25] Q. Fan, P. Hou, C. Choi, T. S. Wu, S. Hong, F. Li, Y. L. Soo, P. Kang, Y. Jung, Z. Sun, *Adv. Energy. Mater.* **2019**, *10*, 1903068.
[26] J. Yang, Z. Qiu, C. Zhao, W. Wei, W. Chen, Z. Li, Y. Qu, J. Dong, J. Luo, Z. Li, Y. Wu, *Angew. Chem.-Int. Edit.* **2018**, *57*, 14095.
[27] Y. Cheng, S. Zhao, H. Li, S. He, J.-P. Veder, B. Johannessen, J. Xiao, S. Lu, J. Pan, M. F. Chisholm, S.-Z. Yang, C. Liu, J. G. Chen, S. P. Jiang, *Appl. Catal. B* **2019**, *243*, 294.
[28] K. Jiang, S. Siahrostami, T. Zheng, Y. Hu, S. Hwang, E. Stavitski, Y. Peng, J. Dynes, M. Gangisetty, D. Su, K. Attenkofer, H. Wang, *Energy Environ. Sci.* **2018**, *11*, 893.

[29] Z. Chen, X. Zhang, W. Liu, M. Jiao, K. Mou, X. Zhang, L. Liu, *Energy Environ. Sci.* **2021**, *14*, 2349.
[30] X. Wang, Y. Wang, X. Sang, W. Zheng, S. Zhang, L. Shuai, B. Yang, Z. Li, J. Chen, L. Lei, N. M. Adli, M. K. H. Leung, M. Qiu, G. Wu, Y. Hou, *Angew. Chem. Int. Ed.* **2021**, *60*, 4192.
[31] S.-G. Han, D.-D. Ma, S.-H. Zhou, K. Zhang, W.-B. Wei, Y. Du, X.-T. Wu, Q. Xu, R. Zou, Q.-L. Zhu, *Appl. Catal. B* **2021**, *283*, 119591.
[32] Z. Zhang, C. Ma, Y. Tu, R. Si, J. Wei, S. Zhang, Z. Wang, J.-F. Li, Y. Wang, D. Deng, *Nano Research* **2019**, *12*, 2313.
[33] J. Gu, C.-S. Hsu, L. Bai, H. M. Chen, X. Hu, *Science* **2019**, *364*, 1091.
[34] F. Pan, Hanguang Zhang, Kexi Liu, David Cullen, Karren More, Maoyu Wang, Zhenxing Feng, Guofeng Wang, Gang Wu, Y. Li, *ACS Catal.* **2018**, *8*, 3116.
[35] C. Zhang, S. Yang, J. Wu, M. Liu, S. Yazdi, M. Ren, J. Sha, J. Zhong, K. Nie, A. S. Jalilov, Z. Li, H. Li, B. I. Yakobson, Q. Wu, E. Ringe, H. Xu, P. M. Ajayan, J. M. Tour, *Adv. Ener. Mater.* **2018**, *8*, 1703487.
[36] H. Chen, X. Guo, X. Kong, Y. Xing, Y. Liu, B. Yu, Q.-X. Li, Z. Geng, R. Si, J. Zeng, *Green Chemistry* **2020**, *22*, 7529.
[37] H. Zhang, J. Li, S. Xi, Y. Du, X. Hai, J. Wang, H. Xu, G. Wu, J. Zhang, J. Lu, J. Wang, *Angew. Chem. Int. Ed.* **2019**, *58*, 14871
[38] Z. Geng, Y. Cao, W. Chen, X. Kong, Y. Liu, T. Yao, Y. Lin, *Appl. Catal. B* **2019**, *240*, 234.
[39] X. Wang, Z. Chen, X. Zhao, T. Yao, W. Chen, R. You, C. Zhao, G. Wu, J. Wang, W. Huang, J. Yang, X. Hong, S. Wei, Y. Wu, Y. Li, *Angew. Chem. Int. Ed.* **2018**, *57*, 1944.
[40] J. Feng, H. Gao, L. Zheng, Z. Chen, S. Zeng, C. Jiang, H. Dong, L. Liu, S. Zhang, X. Zhang, *Nat. Commun.* **2020**, *11*, 4341.
[41] B. Zhang, J. Zhang, J. Shi, D. Tan, L. Liu, F. Zhang, C. Lu, Z. Su, X. Tan, X. Cheng, B. Han, L. Zheng, J. Zhang, *Nat. Commun.* **2019**, *10*, 2980.
[42] H. Yang, Y. Wu, G. Li, Q. Lin, Q. Hu, Q. Zhang, J. Liu, C. He, *J. Am. Chem. Soc.* **2019**, *141*, 12717.
[43] F. Yang, X. Mao, M. Ma, C. Jiang, P. Zhang, J. Wang, Q. Deng, Z. Zeng, S. Deng, *Carbon* **2020**, *168*, 528.
[44] W. Zheng, J. Yang, H. Chen, Y. Hou, Q. Wang, M. Gu, F. He, Y. Xia, Z. Xia, Z. Li, B. Yang, L. Lei, C. Yuan, Q. He, M. Qiu, X. Feng, *Adv. Funct. Mater.* **2019**, *30*, 1907658.
[45] Z. Chen, K. Mou, S. Yao, L. Liu, *ChemSusChem* **2018**, *11*, 2944.
[46] M. Fang, X. Wang, X. Li, Y. Zhu, G. Xiao, J. Feng, X. Jiang, K. Lv, Y. Zhu, W. F. Lin, *ChemCatChem* **2020**, *13*, 603.
[47] N. Wang, Z. Liu, J. Ma, J. Liu, P. Zhou, Y. Chao, C. Ma, X. Bo, J. Liu, Y. Hei, Y. Bi, M. Sun, M. Cao, H. Zhang, F. Chang, H.-L. Wang, P. Xu, Z. Hu, J. Bai, H. Sun, G. Hu, M. Zhou, *ACS Sustain. Chem. Eng.* **2020**, *8*, 13813.
[48] F. Yang, P. Song, X. Liu, B. Mei, W. Xing, Z. Jiang, L. Gu, W. Xu, *Angew. Chem. Int. Ed.* **2018**, *57*, 12303
